# Saline systems of the Great Plains of western Canada: an overview of the limnogeology and paleolimnology

**DOI:** 10.1186/1746-1448-1-10

**Published:** 2005-11-18

**Authors:** William M Last, Fawn M Ginn

**Affiliations:** 1 Department of Geological Sciences, University of Manitoba Winnipeg, R3T 2N2 Canada; 2 Department of Microbiology, University of Manitoba, R3T 2N2 Canada

## Abstract

In much of the northern Great Plains, saline and hypersaline lacustrine brines are the only surface waters present. As a group, the lakes of this region are unique: there is no other area in the world that can match the concentration and diversity of saline lake environments exhibited in the prairie region of Canada and northern United States. The immense number of individual salt lakes and saline wetlands in this region of North America is staggering. Estimates vary from about one million to greater than 10 million, with densities in some areas being as high as 120 lakes/km^2^.

Despite over a century of scientific investigation of these salt lakes, we have only in the last twenty years advanced far enough to appreciate the wide spectrum of lake types, water chemistries, and limnological processes that are operating in the modern settings. Hydrochemical data are available for about 800 of the lake brines in the region. Composition, textural, and geochemical information on the modern bottom sediments has been collected for just over 150 of these lakes. Characterization of the biological and ecological features of these lakes is based on even fewer investigations, and the stratigraphic records of only twenty basins have been examined.

The lake waters show a considerable range in ionic composition and concentration. Early investigators, concentrating on the most saline brines, emphasized a strong predominance of Na^+ ^and SO_4_^-2 ^in the lakes. It is now realized, however, that not only is there a complete spectrum of salinities from less than 1 ppt TDS to nearly 400 ppt, but also virtually every water chemistry type is represented in lakes of the region. With such a vast array of compositions, it is difficult to generalize. Nonetheless, the paucity of Cl-rich lakes makes the northern Great Plains basins somewhat unusual compared with salt lakes in many other areas of the world (e.g., Australia, western United States). Compilations of the lake water chemistries show distinct spatial trends and regional variations controlled by groundwater input, climate, and geomorphology. Short-term temporal variations in the brine composition, which can have significant effects on the composition of the modern sediments, have also been well documented in several individual basins.

From a sedimentological and mineralogical perspective, the wide range of water chemistries exhibited by the lakes leads to an unusually large diversity of modern sediment composition. Over 40 species of endogenic precipitates and authigenic minerals have been identified in the lacustrine sediments. The most common non-detrital components of the modern sediments include: calcium and calcium-magnesium carbonates (magnesian calcite, aragonite, dolomite), and sodium, magnesium, and sodium-magnesium sulfates (mirabilite, thenardite, bloedite, epsomite). Many of the basins whose brines have very high Mg/Ca ratios also have hydromagnesite, magnesite, and nesquehonite. Unlike salt lakes in many other areas of the world, halite, gypsum, and calcite are relatively rare endogenic precipitates in the Great Plains lakes. The detrital fraction of the lacustrine sediments is normally dominated by clay minerals, carbonate minerals, quartz, and feldspars.

Sediment accumulation in these salt lakes is controlled and modified by a wide variety of physical, chemical, and biological processes. Although the details of these modern sedimentary processes can be exceedingly complex and difficult to discuss in isolation, in broad terms, the processes operating in the salt lakes of the Great Plains are ultimately controlled by three basic factors or conditions of the basin: (a) basin morphology; (b) basin hydrology; and (c) water salinity and composition. Combinations of these parameters interact to control nearly all aspects of modern sedimentation in these salt lakes and give rise to four 'end member' types of modern saline lacustrine settings in the Great Plains: (a) clastics-dominated playas; (b) salt-dominated playas; (c) deep water, non-stratified lakes; and (d) deep water, "permanently" stratified lakes.

## Introduction

*"The scientific exploration of North American salt lakes was relatively slow off the mark." *[1]

The geoenvironmental examination of lakes had its origin during the latter part of the nineteenth century with classic works on the Great Basin lakes of western United States [2-4] and central Europe [5,6]. Although these early landmark studies were directly responsible for the formulation and development of many of our currently-accepted ideas about such well known geological processes as turbidity flow, evaporative concentration and mineral precipitation, and deltaic/shoreline sedimentation, progress on the study of lacustrine sediments, as a distinct sedimentary entity, throughout most of the twentieth century was slow. As recent as just a few decades ago, the status of lake sediment research was equated to that of the hole in a doughnut [7].

Interest in lacustrine geological processes and lake deposits increased dramatically beginning in the 1970's [8-10]. This geoscientific involvement with lakes is attributed to two factors: (i) the recognition that lake sediments provide a source of valuable industrial minerals and fossil fuels [11-14], and (ii) the increased use of inorganic components of lake sediments to monitor pollution, decipher environmental changes, and deduce past climatic and hydrological conditions [15-18].

Geolimnology, a term introduced by Professor J. T. Teller [19,10], is the study and interpretation of physical, biological, geochemical, and hydrogeological processes in lakes and the sedimentological records of lacustrine basins. During the past fifteen years there has been rapid advance in our understanding of the physical and chemical processes operating in lakes and how these processes apply to the stratigraphic sequences preserved in lacustrine basins [20-23]. It is now generally accepted that probably no other continental setting has as much to offer in terms of potential significant contributions to the Earth sciences as the lake environment [24-27]. Paralleling this dramatic increase in growth in geolimnology, the subdiscipline of paleolimnology has also seen an explosion of interest and widespread application [28-30]

**Figure 1:**
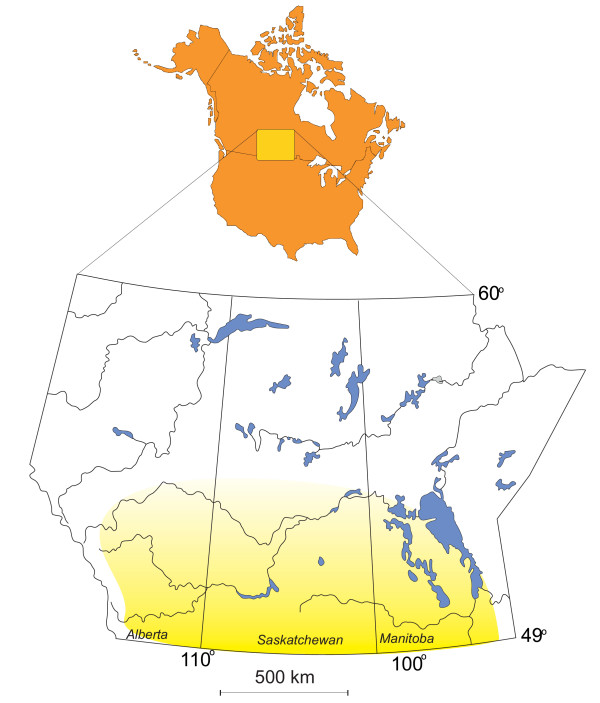
**Northern Great Plains, western Canada**. Location map of the northern Great Plains of western Canada (yellow shading) and the provinces of Manitoba, Saskatchewan, and Alberta.

In this paper we wish to introduce and provide an overview of recent advances in our geolimnological understanding of the wide assortment of modern lacustrine environments in the northern Great Plains of western Canada (Figure 1). We hope this overview will also help establish a framework for future limnological, limnogeological, and paleolimnological research efforts on the Holocene sedimentary records preserved in lakes in this large geographic region of North America. Within the space limitations of this paper it is important to note that emphasis is placed on *saline *lakes and *saline *lake sediments, and on *extant *lakes and their sediment records. Comprehensive reviews of the biological aspects of the lakes in this region are provided elsewhere [31-39]. Likewise, the sedimentology, chronology, history and development of extinct (mainly glacial and proglacial) lakes and wetlands are summarized in numerous other publications [e.g., [40-45]]. Unfortunately, with the notable exception of Lake Winnipeg [46-48], there has been little geolimnological research on the few but interesting freshwater basins.

## Setting and physical background for geolimnology on the northern Great Plains

*"...in the central part of the continent there is a region, desert, or semi-desert in character, which can never be expected to become occupied by settlers..." *[49].

The northern Great Plains is a land of many contrasts: rolling prairies, deeply incised river valleys, flat, featureless lake plains, sand hills and dunes, hummocky and hilly topography, well-treed uplands and barren, shade-less, wind-swept plains. Although the physical, geological, and climatic setting of the region has been summarized in many other papers and volumes (e.g., [50-60]), it is useful to emphasize here several of the most important characteristics of the region that impart the distinct and often unique geolimnological features, namely geomorphology, climate, and geology.

### Geomorphology

*"A great untimbered, level, dried-up sea of land." *[61]

**Figure 2:**
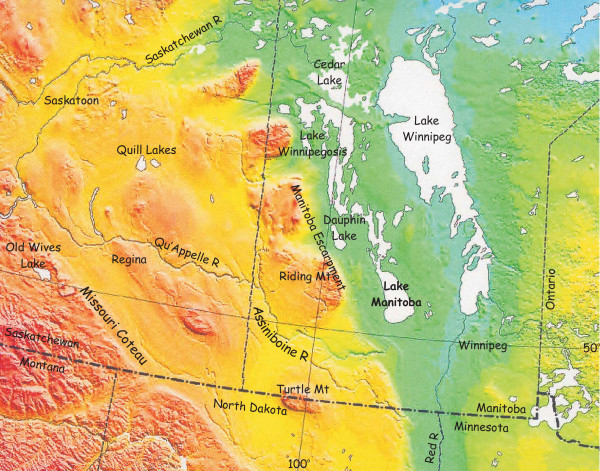
**Eastern portion of northern Great Plains, western Canada**. Shaded relief topography map of the eastern portion of the northern Great Plains of western Canada showing the major geomorphic features and lakes identified and discussed within the text.

**Figure 3:**
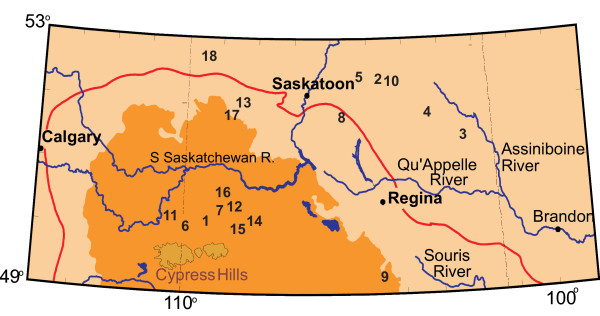
**Western portion of northern Great Plains, western Canada**. Map of the western portion of the northern Great Plains of western Canada. The numbers refer to salt lakes identified or discussed within the text. 1: Ingebright Lake; 2: Deadmoose Lake; 3: Crater Lake; 4: Howe Lake; 5: Muskiki Lake; 6: Bitter Lake; 7: Freefight Lake; 8: Little Manitou Lake; 9: Ceylon Lake; 10: Waldsea Lake; 11: Chappice Lake; 12: Snakehole Lake; 13: Whiteshore Lake; 14: Vincent Lake; 15: Verlo Lake; 16: Corral Lake; 17: Lydden Lake; 18: Metiskow Lake. The brown shaded area (excluding the Cypress Hills) is the driest part of the region, defined by the Brown Chernozemic Soil type. The red line delineates the Palliser Triangle, a region defined by John Palliser during his 19th century expeditions and approximately coinciding with the transition zone between grassland (prairie) and aspen parkland vegetation.

The northern Great Plains physiographic province of Canada stretches from the Precambrian Shield immediately east of Winnipeg, Manitoba, westward for about 1600 km to the Foothills of the Rocky Mountains, and northward some 500 km from the United States-Canada border (Figures 1, 2, and 3). This region is characterized by hummocky to gently rolling topography interspersed with numerous deep, often terraced valleys that have been cut by glacial meltwater.

Within the region, locally separate and distinct sub zones or smaller geomorphological units can be identified. The low (<300 m elevation), often swampy and wetland area of the Manitoba Lowland extends westward from the Precambrian Shield to the Manitoba Escarpment. This area contains the largest lakes in the Great Plains: Lakes Winnipeg, Manitoba, Winnipegosis, Cedar, and Dauphin (Figure 2). With their large drainage basins, generally high sedimentation rates, and early association with extinct giant proglacial lakes, the sediments in these basins reveal long but exceedingly complex developmental histories that are affected by tectonics, evolving landscapes, variable fluvial inputs and regional climate fluctuations.

**Figure 4:**
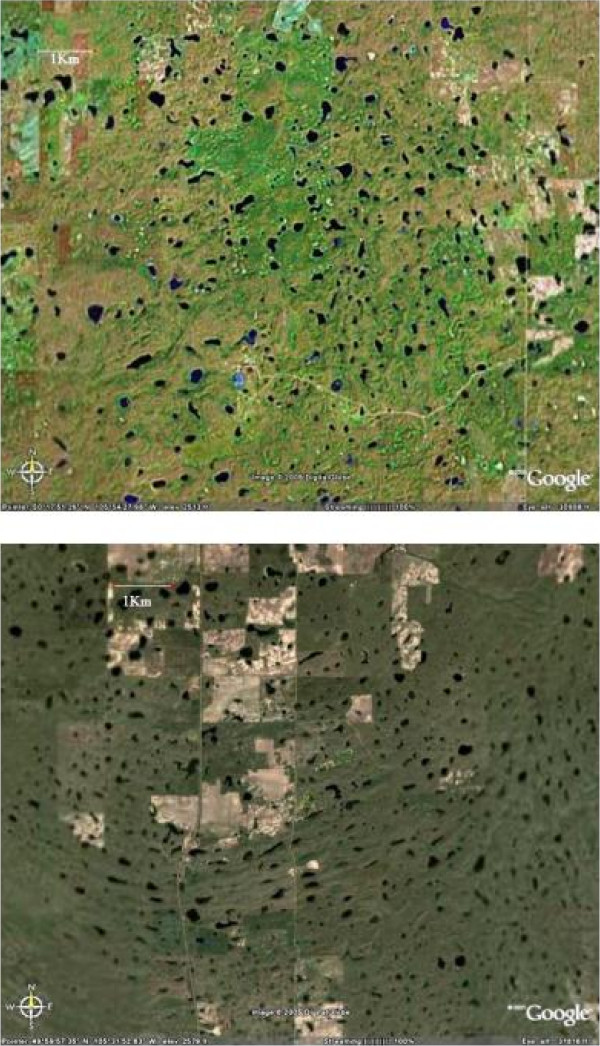
**Examples of high density of saline lake basins in the northern Great Plains**. Top: An aerial image of southern Saskatchewan (area shown approximately 135 km^2^) showing the large number of small lake basins. Bottom: An aerial image of a section of the Dirt Hills area in southern Saskatchewan showing a large number of lakes associated with the hummocky topography of this ice-thrust moraine feature.

Extending westward from about the Manitoba border is the Saskatchewan Plains region or sometimes referred to as the Second Prairie Level. This is a large area of gentle relief between about 450 and 700 m elevation containing a very large number of mainly small and shallow lakes (Figure 4). Estimates range from 4 to 10 million lakes and wetlands in this region [62,39] and densities as high as 90–120 lakes km^-2 ^in some localities [63,64]. The Saskatchewan River Lowlands in the east give way to the greater topographic diversity of the Central Saskatchewan Plains and the various Uplands areas.

**Figure 5:**
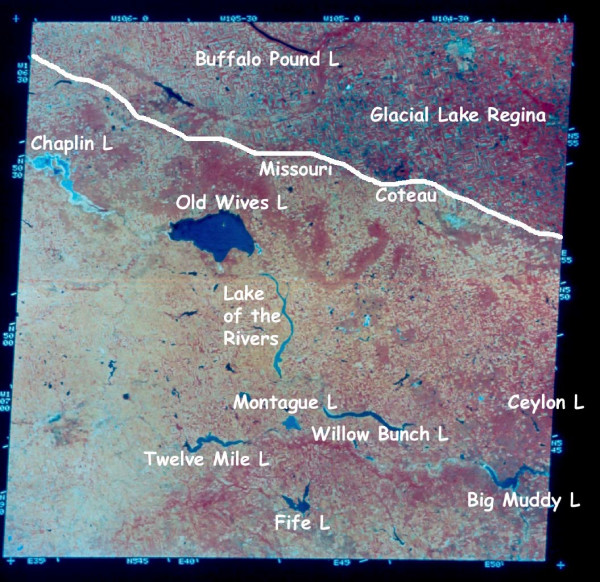
**Landsat image of southern Saskatchewan**. Landsat image of a portion of southern Saskatchewan showing the Missouri Coteau immediately west of glacial Lake Regina. Nearly all lakes show in this image are saline. Note the abundance of lakes west of the Coteau and the strong dominance of riverine basin morphologies in this area of the northern Great Plains.

Separating the Second Prairie Level from the western portion of the geomorphic province is the Missouri Coteau and its eastward-facing edge, the Missouri Escarpment. The Missouri Coteau is a distinct, 50 to 100 km wide band of knob and kettle topography that extends for over 1200 km through the Great Plains from central South Dakota northwestward into west-central Saskatchewan [65]. The terrain in this region is rougher and the elevation (550 to 1400 m) greater than in the Central Saskatchewan Plains to the east. In some areas "badlands" topography has developed with local relief being as much as 150 m. The Coteau is an important geomorphic feature of the Plains and contains many brackish and to hypersaline lakes (Figure 5).

Finally, the Alberta Plains (or Third Prairie Level) continue westward from western Saskatchewan to meet the Foothills of the Rocky Mountains. Although this western part of the Great Plains contains relatively fewer lake basins, nonetheless, several of the best-studied Holocene lacustrine stratigraphic sequences occur here.

### Climate

*"The Great Plains are a region of temperature and precipitation extremes: a decidedly continental climate that nearly defies generalization." *[52]

Long-term (>100-year) temperature, precipitation, and other climatic records for the region exist for Winnipeg, Brandon, Indian Head, Regina, Swift Current, Medicine Hat Edmonton, and Calgary, with shorter records for some 200 other climatic stations in the northern Great Plains. In general terms, the northern Great Plains experience a cold continental, sub-humid to semi-arid steppe climate. Stable, high pressure continental and Arctic air masses dominate during the winter months giving the region its characteristic cold, clear weather. Most of the region is south of the mean path of winter low pressure systems, but pressure and temperature gradients associated with these systems often lead to the area being influenced by high winds. Continental Arctic and Polar air masses dominate the summer weather, resulting in generally warm and dry conditions. Mean daily temperature during January over most of the region is about -18°C and during July it is 19°C; the mean annual temperature shows a narrow range from 1.1°C to 2.9°C [66]. However, the most important characteristic of the region in terms of temperature is its extreme variability. There are wide variations in temperature between seasons, between years, and between day and night. This temperature variability has a significant impact on many chemical and physical aspects and processes of the lakes in the region.

In addition to temperature, another important climatic factor influencing the geolimnology of the region is the high evaporation to precipitation ratio. The region receives about 40 cm of precipitation per year, whereas as much as 1.5 m of water can be lost annually through evaporation from open water bodies [66]. This annual moisture deficit is one of the major variables that help to impart and control the characteristically high salinities of water in most of the lakes.

Wind is also important in dictating the processes operating in the lakes. Although most (but not all) of the lakes form a winter ice cover, during much of the ice-free season, the average wind speed is moderate to high and mainly from the west and southwest directions. In addition to greatly aiding evaporation of water from the lakes, wind plays a major role in current and wave generation and, hence, sediment deposition and erosion [e.g., [67]]. Wind has also been shown to cause significant local variation in sedimentary facies patterns within basins, and can be an important agent of transport of clastic sediment and salts into or out of the lakes [68-70].

### Hydrology and Geology

*"We passed during the day many salt lakes, fringed round the edges with thick encrustations of salt, highly indicative of the rapid evaporation that takes place in these arid regions. *(J. Palliser, October, 1857, on the area north and west of present-day Lake Diefenbaker) [71]

**Figure 6:**
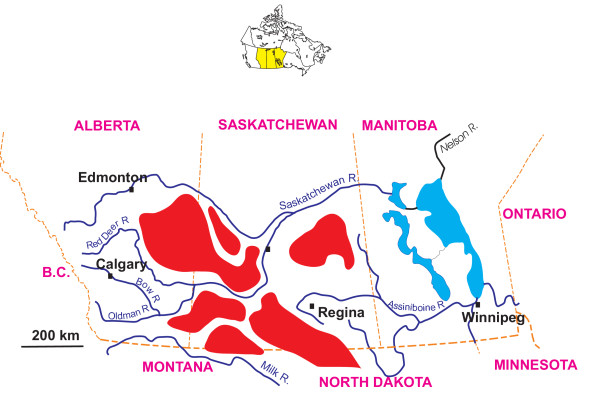
**Closed basins, northern Great Plains**. Map showing major drainage systems of the northern Great Plains of western Canada and the areas of closed (internal) drainage.

Probably the most significant factor influencing the nature, distribution, and sedimentary characteristics of the lakes is the presence of large areas of internal drainage in the northern Great Plains. Because of its lack of integrated drainage, the Missouri Coteau contains many individual closed basins. Further large areas of internal drainage also exist east of Saskatoon, west and north of Swift Current and in eastern and central Alberta (Figure 6). Together, these basins comprise one of the largest and best studied areas of endorheic drainage in North America [72-77].

The lack of integrated drainage patterns throughout such a large region makes precise definition of the various watersheds and drainage divides somewhat difficult. The areas of the northern Great Plains that are not characterized by closed basins are drained by three major river systems. The Saskatchewan system originates in western Alberta and, together with the Qu'Appelle-Assiniboine system, drains southern Saskatchewan to the east into Lakes Manitoba and Winnipeg, and, ultimately, north into Hudson Bay. Runoff in the southern-most parts of Saskatchewan and Alberta is directed south into the Missouri River system and onward to the Gulf of Mexico.

The Canadian Plains region is underlain by nearly horizontal Phanerozoic sedimentary rocks of thicknesses up to 5000 m. The Paleozoic section consists mainly of a series of stacked carbonate-evaporite cycles, whereas the overlying Mesozoic and Cenozoic bedrock is dominantly a sand-shale sequence. Dissolution of the highly soluble Paleozoic evaporites by groundwater has modified the relatively simple structural relationships of the flat-lying formations and has created collapse structures over much of the area [78,79]. Several authors have suggested this evaporite dissolution has provided a source of ions for the many salt lakes of the region [80-85].

The bedrock surface has also been strongly modified by preglacial erosion. By the start of the Quaternary Period a mature, dendritic drainage pattern had been established over much of the northern Great Plains [86-88]. The most important of these fluvial channels are the Hatfield, Tyner, Battleford, Swift Current and Estevan Valleys. In general, today's streams reflect this ancestral pattern, except that much of the upper Missouri River flowed northeastward into Hudson Bay rather than into the Mississippi River basin [89,90]. This ancient dendritic drainage morphology is important in helping localize and channel groundwater and therefore is a major factor in salt lake occurrence in the Prairies.

The bedrock is mantled by unconsolidated Quaternary sediment, which is over 300 m thick in places [41]. These deposits consist of till, fluvial sands and gravels, and lacustrine silts and clays. During deglaciation, meltwater from the retreating glacier carved numerous ice-marginal channels and spillways in this sediment [40,91,92]. Although now abandoned or buried under more recent sediment, these valleys often form modern lake basins. The hydrodynamic properties of the Quaternary fill in the valleys influence to a major degree the character and composition of Holocene sediments in these lacustrine basins by controlling the direction of flow and quantity of groundwater discharge [93-97].

Groundwaters play a pivotal role in the geolimnology of this region. As summarized elsewhere [10,98-108], subsurface water compositions in the region are of several main types. Most of the groundwater in unconsolidated "surficial" aquifers is of low to moderate salinity (<3000 ppm total dissolved solids) and dominated by Ca, Mg, and HCO_3 _ions. In the areas of lowest precipitation, shallow drift groundwater is usually dominated by the SO_4 _ion rather than HCO_3_. The shallow bedrock aquifers (Upper Cretaceous and younger rocks) are mainly Na-HCO_3 _in southern Alberta, Ca-Mg-Na-SO_4 _in Saskatchewan, and Ca-Mg-Na-HCO_3 _in western Manitoba. The deeper Paleozoic and Cenozoic bedrock contains higher salinity water (up to 300 ppt TDS) that is usually dominated by Na and Cl ions. The variable input of groundwater from these sources is one of the most significant factors in dictating the brine composition of the lakes at the surface [109].

## Origin of the lake basins

*"Lakes arise from phenomena that are almost entirely geologic in nature. Once formed, they are doomed. Because of the concave nature of basins, there is a compulsory trend toward obliteration as they fill with sediments... Enormous and deep lakes may be far from death as a result of shoaling, but climatic changes or geologic events leading to desiccation or drainage eventually mark their ends." *[110].

**Figure 7:**
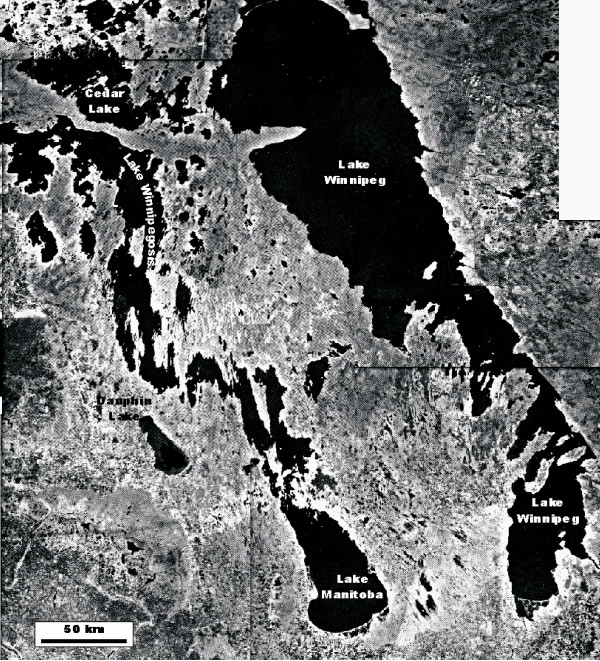
**Large lakes of the Great Plains**. Landsat image showing the Manitoba Lowlands and the presence of large lakes at the extreme eastern side of the northern Great Plains. The Great Plains extend some 1500 km westward from Lake Winnipeg to the foothills of the Rocky Mountains. The large lake basins shown in the image are remnants of glacial Lake Agassiz.

Like the vast majority of lacustrine basins in north-temperate regions of the continent, a glacial origin for most of the lakes of the Canadian Great Plains is evident considering the fact almost the entire area was glaciated during ~23–14 ka. Although only a few lake basins can be attributed to gouging, scraping or scouring action of the glacial ice, many have their origins intimately associated with deglaciation processes. A complex but reasonably clear picture of Late Wisconsinan ice retreat in the Prairie region has emerged over the past several decades [111,41,42]. Ponding of meltwater against the retreating ice margin, due to the regional northward slope and differential isostatic depression, lead to the formation of large proglacial lakes such as Lake Regina, Lake Hind, Lake Saskatchewan, and Lake Agassiz. While few in number, present-day remnants of these proglacial lacustrine basins, such as Lakes Manitoba and Winnipeg in Manitoba (Figure 7) and the Quill Lakes complex in Saskatchewan (Figure 2), are clearly important sources of late Pleistocene and early Holocene paleoenvironmental information. These remnant basins, often simply large but shallow low spots in the glacial deposits, are usually surrounded by old strandlines and glaciolacustrine sediments.

**Figure 8:**
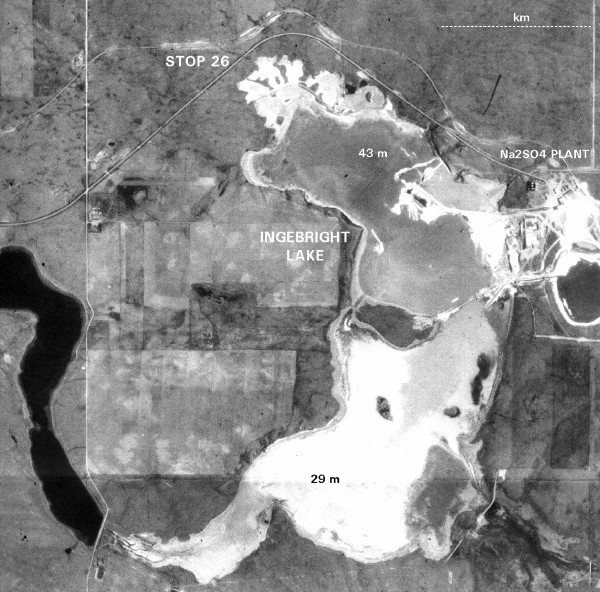
**Ingebright Lake, Saskatchewan**. Aerial photograph of Ingebright Lake, Saskatchewan. This lake host the largest NaSO_4 _deposit in North America (see also Figure 11) and contains in excess of 40 meters of continuous Holocene salt. The depths in meters refer to thickness of salt. North is toward the top of the photo.

As noteworthy as these extensive ice-marginal lakes were, however, only a small number of the millions of extant lakes in the region have been shown to be direct remnants. Instead, the majority of lacustrine basins in the northern Great Plains are the result of stagnant or dead-ice glacial processes which were not sedimentologically directly related to ice-contact precursor lakes. Slow melting of ice buried beneath a thick superglacial drift blanket resulted in creation of a variety of irregular depressions, ice disintegration trenches, kettles, sinkholes, and donuts, as well as the poorly integrated (i.e., topographically closed) drainage which characterizes much of the region [112,10,65,114]. These ice-stagnation basins tend to be small and circular but some have great thicknesses of Holocene and late Pleistocene lacustrine clastics and salts. For example, Ingebright Lake in southwestern Saskatchewan (Figure 8), today a hypersaline playa basin of less than 1 km^2 ^area, contains in excess of 40 m of Holocene salts and clays [115,116]. Similarly, the Deadmoose Lake basin, east of Saskatoon, contains several anomalous troughs and circular depressions up to 50 m deep, which are likely due to ice-block meltout [117,59]

In addition to these hollows on the landscape created by stagnant ice melting, many lake basins in the Great Plains have an obvious fluvial origin as evidenced by their long, linear, riverine morphologies. Like the meltout structures, most of these river-carved basins were created during late Pleistocene deglaciation between about 15 and 11 ka, but some have been shown to occupy older drainage valleys [115].

A number of lake basins in the Plains region owe their existence to several unusual origins. Seismic geophysics and drilling [118,119] confirm that Crater Lake, a small, circular lake about 6 m deep located near Yorkton, Saskatchewan, occupies a collapse chimney created by dissolution of the Prairie Evaporite, some 900 m below the surface. Howe Lake near Wynyard, Saskatchewan, and several other basins and wetlands in North Dakota originated by the process of "hydrodynamic blowout" [[118,120] referenced in [121-123]]. These basins were created when meltwater from the retreating glacier was able to over pressure a shallow groundwater aquifer. The high pressure artesian water exited through a small opening to the surface, in the case of Howe Lake probably a fracture system related to a salt solution-collapse structure. Initially, the extreme pressure was sufficient to expel particles and excavate a basin. Clearly the limnology, sediment composition and overall ecology of the lake in these types of basins are controlled, to a major degree, by the dynamics of the continued groundwater flow and the composition of the groundwater solution. In Howe Lake for example, the groundwater aquifer (sandstones of the Cretaceous Mannville Group) contains freshwater, thus Howe Lake is anomalously fresh despite its closed basin and high evaporation/precipitation ratios. In the case of Kelley Slough, Salt Lake, and Lake Ardoch in North Dakota, the groundwater aquifer contains saline brines and these lakes are therefore anomalously saline, in this case in spite of a relatively humid climatic setting.

Finally, tectonic features associated with glacial thrusting of previously deposited glacial sediments and bedrock are well known in the northern Great Plains. Lake basins can be created both within the irregular ridge and furrow topography of the deformed thrust blocks (see, for example, Figure 13 in [124] and Figure 3 in [125]).

## Economic geology

*"The brine...is ladled into the kettles, and the salt scooped out as it forms, and allowed to remain for a short time to drain before it is packed in birch bark roggins for transportation to Red river, where it commands twelve shilling sterling a bushel.... The brine is very strong. From one kettle two bushels of salt can be made in one day in dry weather." *[126].

**Figure 9:**
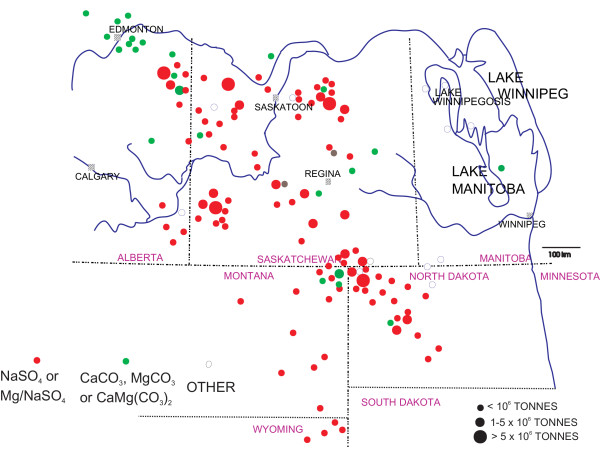
**Salt lake industrial minerals, northern Great Plains**. Map showing the locations and size of lacustrine industrial mineral reserves of the northern Great Plains. The category of reserves listed as 'other' includes halite, silicate, clay, gypsum, and aggregate resources.

The lakes and wetlands of the northern Great Plains serve a great variety of uses. Many studies have documented the importance of these terrestrial environments on surface runoff and flow stabilization, erosion control, waste assimilation, agriculture, irrigation, and wildlife habitat [121,38,39,128-130]. One of the most important economic aspects of the lakes is they are a source of valuable industrial materials, minerals, and compounds (Figure 9).

**Figure 10:**
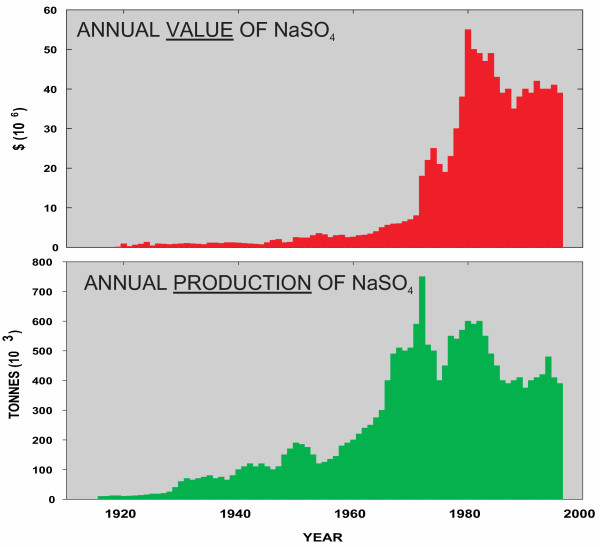
**Sodium sulfate production from salt lakes in the northern Great Plains of western Canada**. Historical changes in value of Na_2_SO_4 _(in Canadian dollars) (upper part of figure) and tonnage (lower part of figure) mined from saline lakes in Alberta and Saskatchewan from 1906 to 2000 [59].

**Figure 11:**
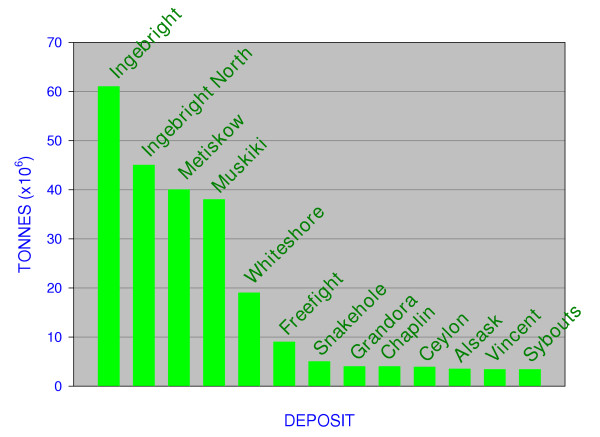
**Na_2_SO_4 _reserves**. Reserves of anhydrous sodium sulfate in the twelve largest lacustrine deposits in the northern Great Plains of western Canada. Ingebright and North Ingebright deposits have been separated but geologically are one contiguous deposit.

Exploitation of the lakes in western Canada probably started well before the arrival of Europeans. Journals and diaries of nineteenth century European settlers commonly refer to Aboriginal use of the lacustrine salts and brines for medicinal purposes, tanning, and food preservation. These salts also provided the basis for several of earliest commercial industrial efforts on the northern Great Plains [126,131]. Large-scale mineral production from the lakes began in 1918 (Figure 10) with the extraction of magnesium and sodium sulfates and carbonates from Muskiki Lake near Saskatoon [80]. Production of anhydrous sodium sulfate (salt cake) from some 20 different lakes (Figure 11) gradually increased over the next five decades to a high of approximately 700,000 tonnes in 1973. Today, the region supplies nearly 50% of the total North American demand for sodium sulfate, with the rest coming from deposits in southwestern United States and as artificial by products from various manufacturing processes. A large increase in the price of salt cake during 1973–1975 (from $15 to $48 tonne^-1) ^and again during 1980–83 (from $62 to $108 tonne^-1^) saw a renewed interest in leasing and mining activities in the region during these periods. Despite softening markets and production declines during the last several years, price stabilization at about $90 tonne^-1 ^has lead to an average of about $30,000,000 worth of sodium sulfate produced annually from the lakes [132,332].

Historically, the two largest uses of sodium sulfate have been in producing kraft paper and allied products, and in the manufacture of detergents [133,85]. More recently, however, the energy industry has been consuming larger amounts of the salt by its use as a conditioner to facilitate fly ash suppression in coal burning power plants [134]. Another new use of salt cake is in the manufacture of potassium sulfate by the reaction of Na_2_SO_4 _with KCl [135-137]. Other potentially significant applications include use in glass, ceramic, and paint manufacture, and in solar energy collectors.

**Figure 12:**
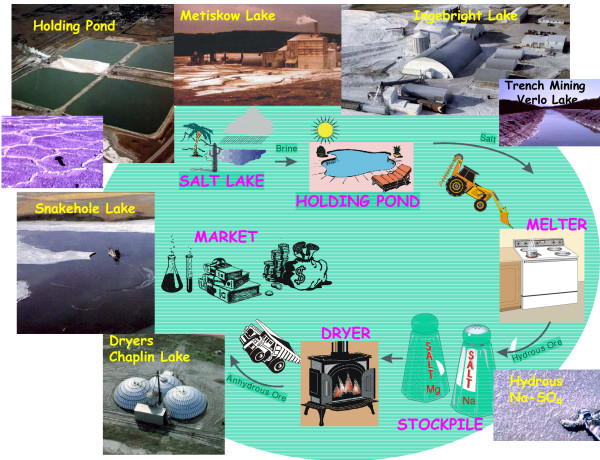
**The lacustrine sodium sulfate industry of the northern Great Plains**. Cartoon showing the industrial processing of lacustrine salts (mainly sodium and magnesium sulfates) from the northern Great Plains of western Canada.

The sodium sulfate industry is based on reserves of three basic types [138,332]: (a) the sodium sulfate that is dissolved in the lake water, (b) the hydrated sodium sulfate mineral mirabilite (Na_2_SO_4 _10H_2_O) which occurs seasonally on the floor of the salt lakes due to precipitation within the overlying water column, and (c) the bedded salts composed mainly of mirabilite and thenardite (Na_2_SO_4_) that make up the Holocene sedimentary fill in some basins. Although each of these three sources has been exploited during the past eighty years, most production today is from the hypersaline lake waters, which are pumped from the basin into holding reservoirs (Figure 12). Upon further concentration by evaporation during summer and then cooling during the fall season, mirabilite (known to the miners as Glauber's salt) is precipitated from the solution. The overlying brine is then drained back into the lake basin, and the salt is removed to stockpiles. Solution mining using hot water and dredge mining of the permanent salt beds have also been used [139].

**Figure 13:**
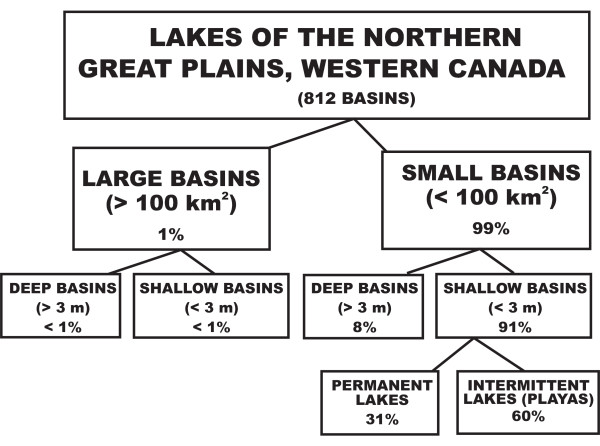
**Salt lake morphology**. Size classification of saline lake basins in the Great Plains of western Canada.

This harvested Glauber's salt (Na_2_SO_4_·10H_2_O) must be dehydrated prior to marketing. The methods for this processing vary considerably [140,133,94]. Most producers simply raise the temperature of the salt to above its fusion point (about 32°C), and then either continue heating to evaporate the water of crystallization or remove the solid anhydrous precipitate from the slurry.

In addition to sodium sulfate, some of the lakes contain marketable amounts of magnesium (in the form of both magnesium sulfates and carbonates [141]), sodium bicarbonate (baking soda [80,142,143,131]), and sodium chloride [131]. Finally, coarse clastics (sands, gravels) deposited on beaches, along shorelines, and in deltas of both proglacial and modern lakes are utilized by many of the urban communities in the region [144-146].

## Size and shape of the lakes

*"The lakes I suppose are not unusual except in numbers alone but if you were able to stand on a great height wherever you are and able to see all the water at once it would still be difficult to find words describing anything but quantity!" *[147]

Basin morphology reveals much about a lake's origin [148] and also exerts a profound influence on the sedimentary processes and resulting spatial distribution of detrital sediments within the lake [149-152,22]. The shape characteristics of the basin similarly help control the distribution of chemical precipitates in lakes [153,154,8].

The lakes of the Plains region exhibit a great range in basin size (from small, less than 1 km^2 ^prairie potholes and kettles to several of the continent's largest basins), shape (from nearly perfectly circular to linear troughs to highly irregular shorelines), and depth (from playas to mean depths of over 25 m). Morphometric details (area, maximum depth, mean depth, shoreline length, shoreline development, volume, and volume development) for over 50 saline lakes in southern Saskatchewan have been described [155,36]. Detailed morphometric information on about 40 lakes in the prairie region of Alberta has been reported in [156]. The saline lacustrine basins of the entire region of the northern Great Plains have also been classified on the basis of size, depth, and degree of permanence [157,158]. Figure 13 shows this classification using an expanded database of about 800 prairie lakes.

Most of the lakes in the northern Great Plains are small and shallow. There are very few basins in the region that can be classified as large (greater than 100 km^2 ^surface area). Four of the six largest lakes are located in the Manitoba Lowland area in the eastern part of the prairies (Figure 7). Lake Winnipeg, the seventh largest lacustrine basin in North America, is also the largest lake in western Canada located off the Precambrian Shield. Lake Manitoba is North America's thirteenth largest lake and Canada's largest saline lake. Of the lakes in southern Saskatchewan and Alberta, two of the largest, Big Quill and Old Wives, are among the six largest inland saline bodies of water on the continent.

Most of the small lakes (less than 100 km^2^) are also shallow. Only about 8% of the basins have mean depths greater than 3 m. However, these small, deep basins are very important. They are attractive targets for paleoenvironmental research because their sediment records often contain undisturbed, finely laminated sequences. It is generally held that deposits in these deeper water basins may be less susceptible to wind and current redistribution and to diagenetic changes brought about by either subaerial exposure or subaqueous chemical fluctuations [159,160]. Several of these small, deep basins are also meromictic, which further enhances their appeal for paleolimnological study [161-163].

Of the many small, shallow salt lakes, one further distinction can be made based on the degree of permanence of the water body. The word *playa*, meaning "beach", "shore" or "coast" in Spanish, has many definitions, synonyms, and, in some cases, contradictory connotations. Most previous researchers on the Great Plains considered a playa to be any low, seasonally flooded but intermittently dry basinal area, and deemed the term to be generally synonymous with ephemeral lake, slough, or wetland. Some also recommend that the term playa be restricted to continental basin settings characterized by an annual net negative water balance (i.e., more water is lost through evaporation and discharge/seepage than is received through all incoming sources) and in which the capillary fringe is close enough to the surface such that evaporation will cause groundwater to discharge to the surface [164,165]. In the northern Great Plains, about half the lakes with mean depths less than 3 m exhibit playa characteristics in that they fill with water during the spring and summer and usually dry completely by late summer. However, not all of these shallow, ephemeral lakes exhibit groundwater discharge.

## Lake water chemistry and properties

*"In this region, there are numerous ponds and small lakes in the hollows among the hills, most of them being more or less brackish or nauseous to the taste from the presence of sulfates of magnesia and soda and other salts. During the dry season of autumn, the water evaporates completely from many of these ponds leaving their beds covered by the dry white salts, which look like snow and are blown about in the wind. Around all the ponds, except those which become completely dry, there is a rank growth of reeds, sedges and grasses, the deep green colour of which forms a strong contrast to the dull grey appearance of the stunted and scanty grass of the hills, which indeed, in many places are almost bare." *[166]

As indicated above, the Great Plains of western Canada contain millions of lakes. Most of these lakes are saline. Throughout much of the region ponded saline and hypersaline brines are the *only *surface waters present. Salinity has a significant impact on the emergent vegetation of the lake's littoral zone and, thus, influences the value of the area as a waterfowl nesting and staging ground. In fact, these lakes, collectively, act as a major breeding ground for 80% of North America's ducks [167,128]. In addition to the importance of this surface water within the realm of wildlife conservation, future agricultural, industrial, and urban development in the Great Plains will likely lead to conflicts and potential environmental problems associated with the lakes in the region [168,169]. Thus, since the first analyses were reported from these lakes over 120 years ago [166], considerable effort has been made to collect water composition data and information relevant to the management of water resources. Historically, both Federal and Provincial agencies have monitored many of the lakes in this region. Numerous compilations of these data, on a regional basis, exist in the literature [170,31,73,156,117,178]. Other important sources of regional water composition information exist for central and eastern Alberta [179,180], southern and central Saskatchewan [181-183], and the Riding Mountain area of western Manitoba [185,186].

**Figure 14:**
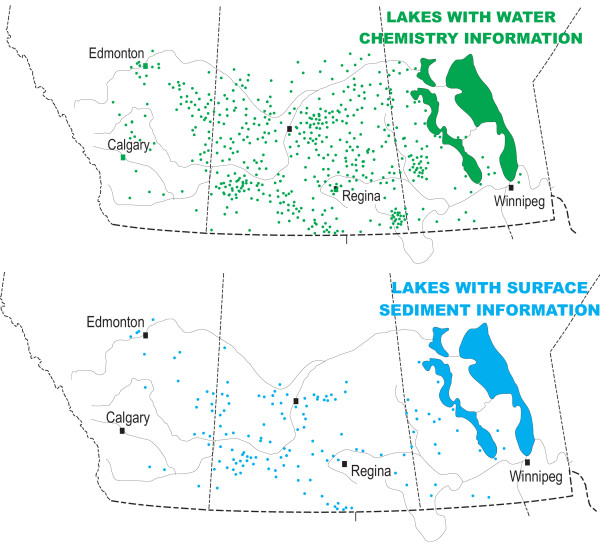
**Salt lake database**. Maps showing the locations of lakes with water chemistry data (upper part of figure) and modern sediment data (lower part of figure); modified from [10, 59, 117, 178].

We now have chemical data from more than 800 of the lakes in the Canadian Great Plains (Figure 14). Although most of these data represent analyses of single samples, some are averages of numerous samples collected over a period of months or years. In general, the larger lakes (e.g., lakes Winnipeg, Manitoba, Quill, etc.) have the longest temporal records, in some cases dating back to the early twentieth century. However, no lake in the Canadian prairies has a *continuous *monitoring record of more than four decades in duration. Of the 800+ lakes for which there are data, 10% are located in Manitoba, 72% in Saskatchewan, and 18% in Alberta.

### Salinity and composition

*"The lakes which fill the hollows are nearly all salt, and even as early in the season of the year, the soil is whitened with salty efflorescence." *[71]

Although a very simple concept, discussion about water salinity can be confusing due to the large variety of methods used to measure this basic parameter [187,10] and a plethora of nomenclature applied (e.g., psu, TDS, ppt, conductivity, molality, g L^-1^, etc.). Biological limnologists often use conductivity as a measure of salinity. Conductivity, or specific conductance, is a measure of the ability of the solution to carry an electrical current: in general, the greater the salinity, the greater the conductivity. However, this salinity-conductivity relationship is not straightforward. It is a function of the specific ions present in the solution, as well as the level of concentration of the ions. For example, a conductivity value of 126 mS_20 _in Bitter Lake, Saskatchewan, which is dominated by sodium and chloride ions, is equivalent to ~100 parts per thousand (ppt or ‰) total dissolved solids (TDS). However, this same conductivity would be measured in a brine having only 75 ppt TDS that was dominated by magnesium and sulfate ions [10,188]. Clearly, the use of conductivity to estimate salinity in lakes having diverse chemical compositions, such as these basins in western Canada, should be avoided despite the inherent convenience of this method.

The abundance of individual ionic components in the water is often reported as weight concentration of the ion in solution (i.e., weight of the dissolved ion in g or mg per kg of solution). This is preferred to using the weight per litre of solution because of the large increase in density of the solution at high salinities. However, care must be exercised in evaluating and comparing published reports using g L^-1 ^units. Unless the actual density of the solution is taken into account by the analyst, the difference between weight/weight versus weight/volume can be significant at elevated salinities [189]. For example, the bottom water of Freefight Lake, which is a deep, meromictic lake in Saskatchewan, is 275 ppt (weight/weight) or 340 g L^-1^.

Investigators involved with appraising the saline lake waters from the perspective of thermodynamics of the aqueous/mineral reactions usually report water composition using traditional chemical concentration nomenclature: molal units (m; moles per kilogram of solvent) or molar units (M; moles per litre of solution) and in equivalents. Equivalents, equivalent weight, or sometimes called combining weight, is the formula weight of the dissolved component divided by its valence charge. Equivalent weights are particularly useful when comparing ionic ratios of one water with another.

In most non-saline aqueous systems, molal and molar units are essential the same. In concentrated solutions, such as those in the lakes of western Canada, differences become significant. Discussions dealing with mineral precipitation/dissolution in the lakes usually evaluate thermodynamic activity of the ions (γ) and ionic strength (I, which is one half the sum of the product of molality times the square of the valence of each ion). In lake waters having less than about 30 ppt TDS, there is relatively little difference between thermodynamic activity and laboratory-derived molality. However, at higher concentrations the electrostatic interactions between the ions greatly reduce their thermodynamic concentration and ionic strength is considerably smaller than molality. Furthermore, like conductivity, γ is also greatly dependent on the specific ions in solution: a 1 m NaCl solution has an ionic strength of 1, whereas I of a solution having the same analytical concentration but dominated by NaSO_4 _is 3 [190].

Nomenclature and terminology also vary widely for the various levels of salinity [191,192,36,10]. Biological limnologists often use the classification scheme of: fresh water (less than 1‰), subsaline (1–3‰), hyposaline (3–20‰), mesosaline (20–50‰), and hypersaline (greater than 50‰). Groundwater researchers usually refer to fresh water as less than 1‰, brackish water as 1–10‰, saline water as 10–100‰, and brine as greater than 100‰. Most geoscientific literature uses: fresh water (less than 3‰), saline (3–35‰), and hypersaline (greater than 35‰).

**Figure 15:**
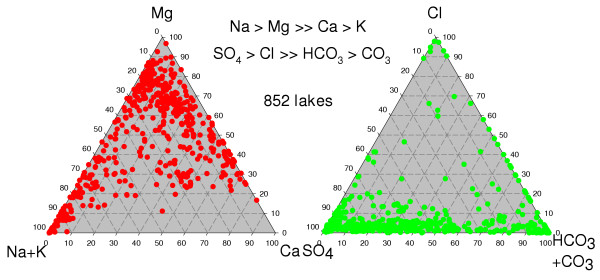
**Brine composition**. Ternary diagrams showing the range of cation and anion composition (eq%) of saline lake waters from the Great Plains of western Canada; modified from [10, 109].

Even though nearly all of the lakes in the Great Plains have similar origins and most are relatively small and shallow, the waters show considerable diversity in terms ionic composition and concentration (Table 1). Early investigators, mainly economic geologists concentrating on the most saline brines, emphasized a strong predominance of Na and SO_4 _in the lakes [80,193]. This importance of sodium and sulfate components in the lakes was similarly recognized by later researchers [73,175]. However, it was not until near the end of the 20^th ^century that the compositional range and degree of diversity of the lakes on a regional basis became obvious. It is now clear that not only is there a complete spectrum of salinities (Figure 15), but also virtually every water chemistry type is represented in lakes of the region [63,109].

**Table 1:** Mean brine composition of saline and hypersaline lakes in selected geographic areas of the northern Great Plains (ionic concentrations in mmol/l; TDS in ppt); modified from [63].

Geographic Area	Ca	Mg	Na	K	HCO_3_	CO_3_	Cl	SO_4_	TDS
Eastern Prairies	4	24	4	1	6	1	2	24	3
Central Saskatchewan	19	149	193	5	7	3	54	251	22
SW Saskatchewan/SE Alberta	12	93	1088	4	96	36	29	1073	80
West-central Saskatchewan and east-central Alberta	3	144	1362	10	268	44	107	1125	102

**Figure 16:**
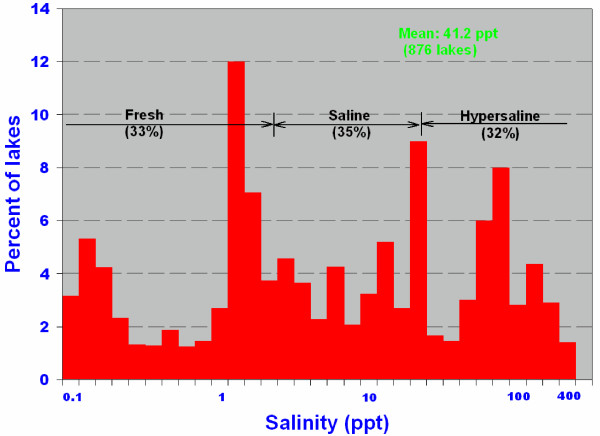
**Salinity of lakes**. Histogram of water salinity of lakes from the Great Plains region of western Canada. Note logarithmic salinity scale; modified from [10, 109].

It is not surprising that the lake waters of the northern Great Plains show such a considerable range in ionic composition and concentration, considering the enormous geographic area and the varying hydrologic, geomorphic, and climatic settings. The lakes range in salinity from relatively dilute water (0.1 ppt TDS) to brines more than an order of magnitude greater than normal sea water (Figure 16). Although it is obviously misleading to generalize by quoting means and averages, the "average" lake water has about 40 ppt TDS and shows: Na>Mg>Ca>K and SO_4_>Cl>HCO_3_>CO_3_.

With such a vast range of salinities, it follows that the concentrations of the individual ionic components also vary greatly. The frequency distributions of Mg, Na+K, Cl, and SO_4 _concentrations in the lake waters tend to be multimodal as opposed to the Ca and HCO_3 _ions which show a much narrower distribution pattern.

Sulfate and carbonate-rich lakes clearly dominate the Great Plains [63,109], comprising over 95% of the total lakes. This paucity of Cl-rich lakes makes the region unusual compared with many other areas of the world (e.g., Australia, western United States). The cation ratios are considerably more diverse, with the abundance of all three major types showing approximately subequal proportions.

**Figure 17:**
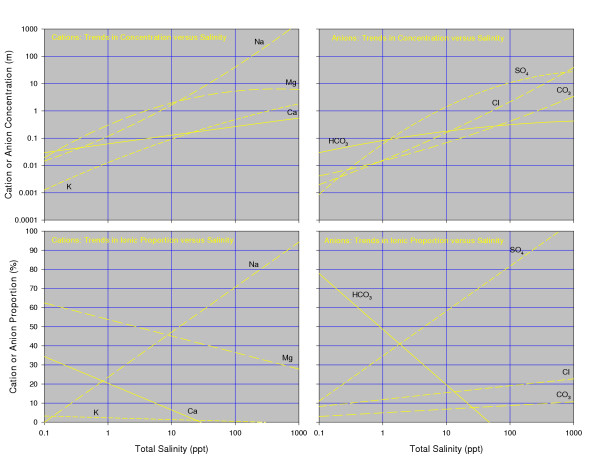
**Ion composition trends with salinity**. Best-fit trend lines showing the change in ionic concentration and ionic proportion with increasing salinity in lakes from the Great Plains region of western Canada. Note logarithmic salinity scale. Modified from [10, 109].

As would be expected, most of the solutes in the lake waters increase in concentration with increasing total salinity (Figure 17). Sulfate and sodium + potassium ions show the most statistically significant correlations with TDS, whereas calcium and carbonate concentrations are less directly related to salinity.

The *proportions *of some of the solutes also show a systematic change with salinity. Sulfate increases in relative ionic proportion from less than 30% equivalents in dilute lakes to generally more than 90% in lakes with more than 10 ppt TDS. Calcium and bicarbonate + carbonate proportions show an inverse relationship with salinity, decreasing from over 70% equivalents in the dilute waters to nearly 5% in lakes with more than 25 ppt TDS.

### Spatial variation

*"Little Manitou lake, near the town of Watrous, contains a salt of slightly different type. The lake is deep and does not evaporate in summer. Therapeutic value is claimed for the waters and it is much used as a summer resort." *[194]

**Figure 18:**
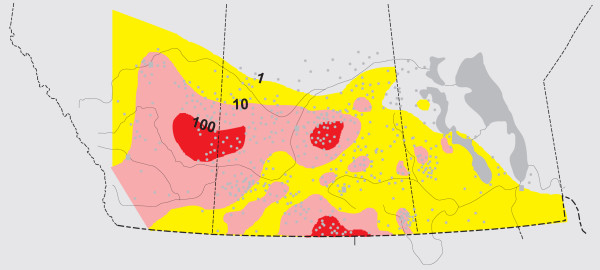
**Regional salinity changes**. Map showing the variation in total dissolved solids of lakes in the northern Great Plains of western Canada. Contour interval is logarithmic; values in ppt TDS; modified from [59, 109].

The relatively uniform distribution of lakes in the Great Plains for which water chemistry data exists permits examination of the ionic contents on a spatial basis similar to that undertaken in the United States portion of the prairies [195,196]. The regional changes in TDS are shown in Figure 18; the results of these regional trend analyses of individual ions are summarized elsewhere: [157,177,84,109]. Lakes with highest Na+K, Mg, and SO_4 _*concentrations *generally occur in the east-central Alberta, west-central and southern Saskatchewan area, whereas lakes with high HCO_3_+CO_3 _and Cl contents are found in central Alberta and western Saskatchewan. Lakes with relatively low *proportions *of Ca and Mg occur in the northern and central parts of the Plains.

### Water chemistry controlling factors: a statistical approach

*"Geology differs from the experimental sciences in that most geological data are fragmentary and are derived from surface manifestation of natural processes that are uncontrolled by the investigator.... So the geologist must make do with the data available, which are seldom those with which he would prefer to work. It is here that statistics may enable him to plan data collection and deduce inferences that are not readily discernable from the raw fragmentary observations that he collects." *[197]

Ion composition and concentration of these lake waters are the result of: (a) a complex interaction between unconsolidated glacial and nonglacial sediments, bedrock, and precipitation/meltwater in the drainage basin, (b) the composition and quantity of groundwater recharge (and discharge) and streamflow in each basin, and (c) a variety of other physical, chemical, and biological processes operating within the water column itself. In general, several different approaches have been taken to help understand the interplay of these major factors controlling surface water chemistry. These geochemical investigative methods include mass balance calculations, thermodynamic equilibrium considerations, and statistical evaluations [198,199,190]. In western Canada specifically, both mass balance and thermodynamic calculations have proved valuable in deciphering many of the intrinsic (i.e., within the drainage basin) processes important in water composition on a local scale [200,157,204]. In contrast to these local studies, on a regional scale, numerous statistical techniques have been successfully applied to help understand the relationships between the water chemistry and extrinsic environmental factors (e.g., climate, bedrock type, geomorphology, till composition [205,206,109,10]). It should be noted that these statistical approaches lack the ability to resolve the often-important *local *conditions and processes; however, they are essential to our overall understanding of the lacustrine geochemical setting of the region as a whole.

One of the most clear-cut ways to analyse the interrelationships within any data set is to examine the linear correlations that exist among the analytical parameters. Not surprisingly, the *concentrations *of Na, Ca and Mg in the brines of these lakes are all significantly positively correlated, as are SO_4 _and Cl. In addition, the ion pairs of Mg-SO_4_, Mg-Cl, and Na-Cl tend to strongly covary. Importantly, the concentrations of Na and SO_4 _do not show statistically significant linear correlation, suggesting different suites of processes affect the abundance of each of these ions. The *proportions *of Ca and HCO_3 _exhibit significant positive covariation, whereas the proportions of Mg and Na, and HCO_3 _and SO_4 _are inversely related.

Using a Q-mode cluster analysis (statistical associations among the *lakes*), it is possible to subdivide the lakes into two major categories [109]: a group of relatively high salinity (greater than 20 ppt TDS) lakes and a group characterized by lower TDS values. In contrast, R-mode analysis (statistical associations among the *geochemical parameters*) indicated the following groups of variables: (a) TDS, Na, and SO_4_; (b) K and Cl; (c) Ca and Mg; and (d) HCO_3 _and CO_3_.

By combining morphological (basin area, maximum depth), geological (bedrock type, depth to bedrock, till type), hydrological (drainage basin area, number of streams entering lake, elevation, groundwater composition), and climatic (mean annual precipitation, evaporation, temperature) variables with the 800-lake water chemistry database, R-mode factor analysis can identify a set of seven statistical factors that explained over 90% of the variance in the data [84,177,109]. The interpretation of these statistical factors is that the most important controls of water composition and concentration on a regional basis are: (a) composition of inflowing groundwater, (b) evaporation/precipitation, and (c) elevation or position of the basin within the drainage basin. Variables related to bedrock type, glacial drift composition, fluvial input, and lake morphology are statistically less important.

### Short-term temporal variation in water chemistry

A major complicating factor in characterizing the geochemistry of the lakes of the northern Great Plains is that many of the basins exhibit playa characteristics. This strong seasonality of water levels gives rise to dramatic changes in both ion concentrations and ratios, as demonstrated by numerous studies [207,175,77,211,68]. For example, Ceylon Lake, a salt-dominated playa in southern Saskatchewan, annually undergoes changes in concentration from about 30 ppt TDS to greater than 300 ppt. This lake also exhibits dramatic fluctuations in ionic ratios on a seasonal basis from a Na-(Mg)-SO_4_-HCO_3 _type in early spring to a Mg-(Na)-Cl-SO_4 _composition by fall. Unfortunately, only a few basins in the northern Great Plains have undergone periodic detailed sampling over a period of years.

### Source of salts

*"Many observations about the occurrence of the salts are valid, but interpretations of their genesis have generally been needlessly complex and unsubstantiated by the observed facts." *[212]

The origin and ultimate source of the major ions in the lakes of the northern Great Plains have been topics of considerable discussion in the scientific literature [85]. Some of the early research suggested that dissolution of the deeply buried Paleozoic evaporites could be a possible source for the dissolved components in the lakes. The occurrence of lacustrine basins (and other geomorphic features) whose origin may be ascribed to collapse of salt-solution chimneys [78,83,79] supports this contention. For example, it has been suggested there is a correlation between the occurrence of the lacustrine sodium sulfate deposits at the surface and the presence and trends of various salt units in the Devonian Prairie Formation in the region [83]. However, others maintain that dissolution of the Prairie Evaporite, assuming no other ionic evolution mechanism, could not contribute waters having ionic ratios compatible with the majority of prairie lakes [157,138].

Indeed, as is clear from mapping of the major sodium sulfate bodies [213,63,178,59] and the brine compositions [84,109] there is no consistent spatial correspondence between the surface features and chemistries and the subsurface Paleozoic rocks in most of the Great Plains region. Nonetheless, it is evident that hypersaline groundwaters from the Paleozoic sequence are important contributors to the salinity in the eastern part of the Plains [214,203,215].

In contrast, relatively shallow Cretaceous and Tertiary bedrock, as opposed to the deep Paleozoic sequence, has been implicated as the source of at least some of the dissolved components in the lakes [80,81,216,201]. Furthermore, many researches [93,94,115,100] have stressed the close association of the more saline lacustrine brines with buried preglacial channels, and have concluded that these buried valleys, often filled with relatively porous and permeable sediments, act as conduits for groundwater supplying dissolved material to the lakes.

Finally, rather than invoking bedrock sources, there is considerable support for the source of the ions being largely the Quaternary deposits within which the lakes are immediately situated [73,212,200,217-221], Although simply defining flow patterns and groundwater dynamics in a natural system characterized by poorly integrated surface drainage, undulating surface relief, and multiple discontinuous permeable zones is exceedingly complex [206,222], on a local basis there is some suggestion that the glacial till is a large soluble salt reservoir which can provide salinity to the shallow groundwater systems and therefore to the lakes [223]. A variety of physicochemical and biochemical reactions, including cation exchange, dissolution of feldspars and detrital carbonates, oxidation of reduced-S mineral species and organic matter, and precipitation of authigenic sulfate, carbonate, and silicate phases in the tills can be documented which support this latter hypothesis. Once a convincing ion source can be attributed to the salt budget of a basin, and assuming an unchanging source water chemistry, it is possible to use the level of salinity in the lake to determine the amount of time required to accumulate the dissolved components in selected closed-basin lakes and wetlands in the region [74-76,224]. From this it is possible to identify the episodes during the Holocene at which the now-closed basins overflowed [76].

## Modern lacustrine sediments and sedimentary processes

### Sediment types

*"The study of sedimentary rocks, as compared with igneous rocks, is somewhat hampered by the poverty of terminology." *[225]

**Figure 19:**
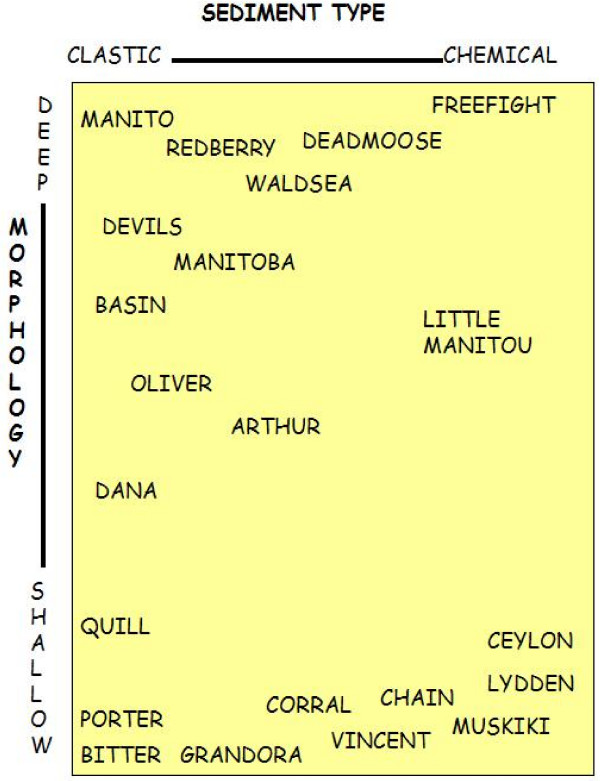
**Sediment type versus morphology**. Range and examples of salt lake types according to morphology and sediment type. Modified from [10, 59 117].

Very few of the millions of saline lake basins in the northern Great Plains have been examined from either a sedimentological or stratigraphic perspective. Of the approximately 800 lakes for which water chemistry has been documented, we have knowledge of the sediments in only about 20% of these. Similar to the early brine composition work, initial sedimentological efforts on these lakes stressed the dominance of sodium sulfate salts, and were directed mainly toward basins with large reserves of economically important industrial minerals. It is now realized that the lakes in the Canadian Great Plains exhibit a complete spectrum of sediment types (Figure 19), from basins dominated by allogenic or clastic material to those in which relatively pure, clastic-free evaporite minerals are forming [157,10].

The modern sediments in most of these lakes consist of mixtures of coarse to finely crystalline salts and organic-rich, silty clays/clayey silts in the offshore portions of the basins, grading to somewhat coarser clastics (sands and silts) in the nearshore areas. Due to their small size and negligible fetch distances, most of the lakes have only a very narrow margin of shoreline/nearshore coarse silts and sands. However, in the larger basins, such as Lake Manitoba or the Quill Lakes, and in lakes with long wind-fetch distances, such as characterize the many linear, riverine lakes, coarse clastics can extend farther out into the basin [226,227]. Because of the paucity of fluvial input to most of the lakes, deltas are rare except in the larger basins such as Lakes Manitoba and Winnipeg. Away from the relatively nearshore areas, however, there is usually little variation in grain size in any of the basins [228,75,46,77], [229-231].

**Figure 20:**
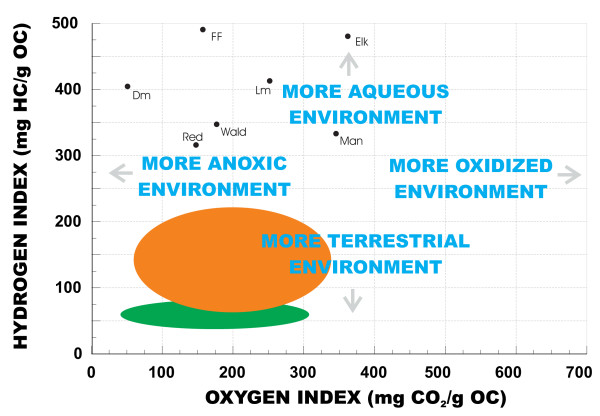
**Rock-Eval analysis of salt lake sediments**. Plot of Hydrogen Index (HI) versus Oxygen Index (OI) values for sediment from saline lakes of the northern Great Plains [modified from [10, 59, 117, 178]. Red = Redberry Lake offshore sediment; FF = Freefight Lake offshore sediment, Elk = Elk Lake (Minnesota [232]) offshore sediment; Lm = Little Manitou Lake offshore sediment; Dm = Deadmoose Lake offshore sediment; Wald = Waldsea Lake offshore sediment; Man = Lake Manitou offshore sediment; green shaded area = samples from playa lakes; Minnesota and the Dakotas [232]; orange shaded area = samples from pedogenic zones in Lake Manitoba [233].

Generally the organic content of the modern sediments is low, although in some basins organic matter can be as much as 45% (by weight) of the total sediment. The organic content of sediments from playa basins and shallow lakes is not significantly different from that of most of the perennial deep water basins. However, the chemical composition of this organic matter can vary significantly. Playa lake sediments in the region show consistently much lower Rock-Eval hydrogen indices (HI) and somewhat higher oxygen indices (OH) relative to both shallow and deep perennial lakes (Figure 20), reflecting a more terrestrial source for the organics in the playas [232,233,10].

### Detrital versus chemical sediments

*"The union of alkaline earths with carbonate and calcium with sulfate are unlikely in Saskatchewan." *[76]

Sedimentologists have long recognized three basic genetic types of sediment in most lacustrine basins; (a) *allogenic *(i.e., detrital): that material derived from weathering and erosion of the soils and bedrock of the watershed and transported to the lake by fluvial, sheetwash, gravity, or aeolian processes; (b) *endogenic*: sediment originating from biological or inorganic processes occurring entirely within the water column of the lake; and (c) *authigenic *(i.e., diagenetic): material resulting from mainly chemical and biological processes occurring once the sediment has been deposited. The endogenic and authigenic fractions are sometimes collectively referred to in a nongenetic sense as 'chemical sediment', as opposed to 'clastic sediment', which is usually dominated by the allogenic fraction. However, use of this latter designation should be avoided when discussing the deposits of saline lakes because of the presence of such things as 'clastic' sediment comprised of fragmented endogenic components, or 'chemical' sediment derived largely by post-depositional diagenetic processes.

The allogenic component in most of the lakes of western Canada consists mainly of a mixture of (in approximate decreasing order of abundance): clay minerals, quartz, carbonates, feldspars, and ferromagnesian minerals. The relative proportion of each of these detrital mineral groups is generally similar to that of the surrounding glacial debris, except sorting by the transporting medium and diagenetic processes operating within the watershed soils can also influence the final detrital mineralogy. The less chemically stable mineral components, such as detrital carbonates and feldspars, are particularly susceptible to loss by weathering processes in the drainage basin [234-237]. Superimposed on this element of chemical stability of the detrital components is the inherent size fractionation of various minerals caused due to physical abrasion and breakage of grains by the action of glacial ice. For example, because the terminal grade for glacier-comminuted dolomite is medium to coarse silt [238,239], the modern sedimentary facies of the larger lakes in the region show a gradation from dolomite-enriched sediment nearshore to dolomite-depleted deposits offshore [46,240,231].

Although the sediments of only a small number of lakes have been examined for detailed clay mineralogy, the most common layered silicates are kaolinite, illite, smectite, and chlorite [228,237,77,230,241,162,233]. A variety of mixed-layer clays, most commonly illite-smectite and illite-chlorite species, have also been reported, but at least some of these interstratifed minerals have been attributed to possible authigenic processes within the lake basins [204,242,233]. In lakes west of the Manitoba Lowland, the sediments are almost completely dominated by smectitic clays, undoubtedly a reflection of the bentonitic Cretaceous shales, which underlie most of the area west of the Manitoba Escarpment. The abundances of kaolinite, illite and smectite in the eastern part of the region tend to be approximately subequal.

The only detrital carbonate minerals identified in the lakes of the Great Plains are dolomite and calcite. In bulk (i.e., non-size fractionated) samples, CaMg(CO_3_)_2 _(dolomite) is usually considerably more abundant than CaCO_3 _(calcite). This is most likely a reflection of the relative abundance of these two minerals in the tills and bedrock of the region, as well as the higher solubility and, hence, lower weathering stability of calcite relative to dolomite [239,229,243,244,231]. Similarly, potassic feldspars are usually more abundant than plagioclase in the lakes due to the partial loss of the relatively unstable Na and Ca feldspars by hydrolysis in the tills and watershed soils.

**Figure 21:**
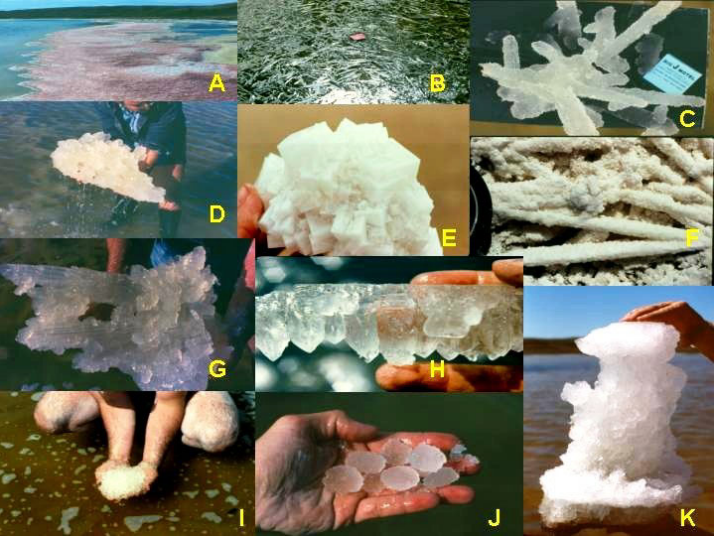
**Endogenic and authigenic salts**. Examples of modern chemical precipitates from the salt lakes of the northern Great Plains of western Canada; modified from [10, 59, 117]. A. Shoreline accumulation of rounded accretionary grains of mirabilite; B. Floating crust of thenardite; depth of brine under the crust is ~40 cm; water temperature is 34°C; C. Large dendritic accumulation of bloedite crystals; D. Large subaqueous accumulation of mirabilite crystals; E. Accumulation of halite crystals; F. Acicular thenardite crystals that have been pseudomorphologically replaced by halite; G. Large aggregate of epsomite crystals; H. Dogtooth crystals of mirabilite overlain by flat-lying bloedite crystals; I. and J. Rounded accretionary grains of mirabilite, termed mirabolites; K. Spring-orifice accumulation of bloedite and mirabilite.

Considering the great range of water compositions in lakes of the Great Plains, it is not surprising there is an equally significant breadth of endogenic and authigenic minerals found in these lakes. In the playas of the Great Plains, there are two main types of endogenic precipitates in the modern sediments: (a) very soluble salts, comprising mainly sodium and magnesium sulfates and carbonates, and (b) sparingly soluble precipitates, including mainly carbonates, sulfates, and silicates (Figure 21). There have now been close to fifty non-detrital minerals identified from the lakes (Table 2). These endogenic and authigenic minerals can also be subdivided according to their genesis and occurrence within the lake: (a) surficial efflorescent crusts and hardgrounds, usually occupying nearshore and seasonally flooded areas; (b) massive and bedded precipitates, most often found blanketing the floor of the basins from shallow marginal zones down to deep central offshore areas; and (c) accumulations of salts and precipitates associated with either subaqueous or subaerial springs.

**Table 2:** Endogenic and authigenic minerals in saline lakes of the northern Great Plains; modified from [10, 59, 116].

Mineral Name	Composition	Occurrence
**Carbonate Minerals**		
Aragonite	CaCO_3_	very common
Artinite	Mg_2_CO_3_(OH)_2_3H_2_O	very rare
Ankerite	Ca(Fe,Mg)(CO_3_)_2_	rare
Benstonite	Ca_7_Ba_6_(CO_3_)_13_	very rare
Calcite	CaCO_3_	very common
Gaylussite	Na_2_Ca(CO_3_)_2 _5H_2_O	rare
Kutnohorite	Ca(Mn,Mg)(CO_3_)_2_	very rare
Magnesite^1^	MgCO_3_	common
Magnesian Calcite	(Mg_x_Ca_1-x_)CO_3_	very common
Minrecordite	CaZn(CO3)_2_	very rare
Protodolomite	CaMg(CO_3_)_2_	common
Zemkorite	Na_2_Ca(CO_3_)_2_	very rare
		
**Sulfate Minerals**		
Arcanite	(K,NH_4_)_2_SO_4_	rare
Bloedite	Na_2_Mg(SO_4_)_2 _4H_2_O	very common
Despujolsite	Ca_3_Mn(SO_4_)_2_(OH)3(H_2_O)	rare
Epsomite	MgSO_4 _7H_2_O	common
Eugsterite	Na_2_Ca(SO_4_)_3 _2H_2_O	very rare
Gypsum	CaSO_4 _2H_2_O	very common
Hexahydrite	MgSO_4 _4H_2_O	common
Kieserite	MgSO_4 _H_2_O	common
Krausite	Fe_2_(SO_4_)_3 _2H_2_O	very rare
Mercallite	KHSO_4_	very rare
Mirabilite	Na_2_SO_4 _10H_2_O	very common
Potassium alum	KAl(SO_4_)_2_12H_2_O	very rare
Thenardite	Na_2_SO_4_	very common
Wattevilleite	Na_2_Ca(SO_4_)_2 _4H_2_O	very rare
		
**Carbonate-Sulfate, Carbonate-Sulfate-Chloride, and Carbonate-Phosphate Minerals**		
Bonshtedite	Na_3_Fe(PO_4_)(CO_3_)	very rare
Bradleyite	Na_3_Mg(PO_4_)(CO_3_)	very rare
Burkeite	Na_4_(SO_4_)CO_3_	very rare
Rapidcreekite	Ca_2_(CO_3_)SO_4 _4H_2_O	very rare
Tychite	Na_6_Mg_2_SO_4_(CO_3_)	very rare
		
**Chloride Minerals**		
Bischofite	MgCl_2 _6H_2_O	Very rare
Halite	NaCl	rare
		
**Nitrate and Borate Minerals**		
Inderborite	CaMgB_6_O_11_H_2_O	rare
Niter	KNO_3_	rare
Soda Niter	NaNO_3_	rare
Nitrobarite	Ba(NO_3_)_2_	rare
		
**Other**		
Pyrite	FeS_2_	common
Ranciete	Ca_0.75_Mn_4_O_9_·3(H_2_O)	very rare
Sepiolite	Mg_4_Si_6_O_15_(OH)_2_6H_2_O	very rare

^1^Includes hydromagnesite and psuedohydromagnesite.

Distinguishing endogenic lacustrine minerals from those formed by diagenetic processes after the sediment has been deposited (i.e., authigenic) is often an exceedingly difficult task. Many times, detailed petrographic and geochemical studies are required to convincingly demonstrate the precise origin for the specific minerals. To date, few lakes and lacustrine sequences in western Canada have been examined in this amount of detail. Although a complete discussion of the genesis (viz. endogenic versus authigenic) of the minerals listed in Table 2 is beyond the scope of this paper, some of the more common or noteworthy occurrences will be summarized briefly.

Among the endogenic and authigenic carbonate minerals identified in these lakes, aragonite, magnesian calcite (i.e., hi-Mg calcite), and dolomite are the most common. Inorganic precipitation of carbonate minerals due to thermodynamic supersaturation is very common in lakes of all types on a global basis [245,246,8-10]; the Great Plains region is no exception. Nearly all of the 131 lakes that were surveyed in the early 1980's were found to be strongly supersaturated with respect to these carbonate minerals [157]. Supersaturation and precipitation of carbonates can take place for a variety of reasons, including photosynthetic uptake of CO_2 _and consequent increase in pH, concentration changes brought about by evaporation or dilution, temperature changes, and mixing of brines of different compositions. In most of the lakes of the Great Plains, carbonate mineral supersaturation is due mainly to, but not exclusively, the seasonal uptake of CO_2 _by primary organic productivity.

The specific carbonate mineral to be precipitated from the supersaturated solution is controlled mainly by the cations in solution (in particular the ratio of Mg to Ca in the water [247,239]). The elevated Mg/Ca ratios that characterize the lake waters of the Great Plains give rise to a carbonate mineral assemblage dominated by aragonite (the orthorhombic form of CaCO_3_) and Mg-bearing carbonates, such as dolomite, magnesite, huntite and Mg-calcite.

The discovery of non-detrital dolomite in the lakes of the Great Plains has been an important contribution to our understanding of the genesis of this economically important mineral [249]. Dolomite formation in the sedimentary realm is a subject of long-standing interest and study. Probably no other mineral or sedimentary rock has attracted as much speculation regarding its genesis as dolomite [248-250]. The "dolomite problem" has been summarized in many recent reviews [251-254] and in most sedimentary geology textbooks. In essence, the dolomite problem is the enigma that the mineral dolomite (CaMg(CO_3_)_2_) is a very common component of ancient rocks, but is very rare in modern and Holocene sediment. It does not appear to occur as a primary precipitate from marine water of normal salinity and composition, nor cannot be readily synthesized in the laboratory at low temperatures and pressures.

The occurrence of very early diagenetic (i.e., penecontemporaneous) dolomite in the surficial offshore sediments of Devils Lake in northeastern North Dakota [255,256] was one of the earliest documented examples of lacustrine dolomite formation. This occurrence of dolomite also emphasized that dolomitization could take place in solutions of *moderate *salinities (~20 ppt TDS) and in water with high sulfate contents. Others, working mainly in highly evaporitic marginal marine settings, have suggested that dolomite formation is favoured by elevated salinities (greater than 35 ppt TDS) and greatly reduced SO_4 _levels [257-259].

Modern primary and early diagenetic dolomite occurs in numerous lakes in the Canadian Great Plains [249]. Waldsea and Deadmoose Lakes are two adjacent, meromictic saline lakes located in central Saskatchewan. Two distinct types of non-detrital dolomite have been identified in these lakes [260,161,163]. Most is very finely crystalline with subhedral to anhedral crystallites forming aggregates of about 2 μm in diameter. This dolomite, like most Holocene dolomite, is poorly crystalline and poorly ordered (ordering refers to the regular alteration of the layers of cations and anions within the crystal structure), but has a composition close to that of the ideal, stoichiometric mineral (i.e., Ca=Mg). The other type is also poorly crystalline, but is considerably enriched with calcium: Ca(Ca_0.2_Mg_0.8_)(CO_3_)_2_. Like that of Devils Lake, the dolomite in both Waldsea and Deadmoose is forming subaqueously as a primary inorganic precipitate in sulfate-rich waters.

Another important occurrence of dolomite is in Freefight Lake, a 25 m deep, hypersaline meromictic lake in southwestern Saskatchewan. Preliminary sedimentological examination of this lake [261,204,242] indicates a considerable range of formative environments and dolomitization processes. Calcian (i.e., Ca-enriched) dolomite occurs in the deep-water, offshore sediments and also in the subaerially exposed mudflats surrounding the basin. Within the mudflat sediments, it is often associated with aragonite and Mg-calcite, and petrographic evidence suggests it may be an early diagenetic product forming in response to the strong evaporative flux experienced on the mudflats during the ice-free season. In contrast, in the organic-rich, highly reducing offshore sediments, dolomite is associated with a variety of soluble sulfate salts and pyrite. It is forming at the sediment-water interface as a primary precipitate in response to the increased alkalinity brought about by sulfate reduction in the anoxic monimolimnion of the lake.

**Figure 22:**
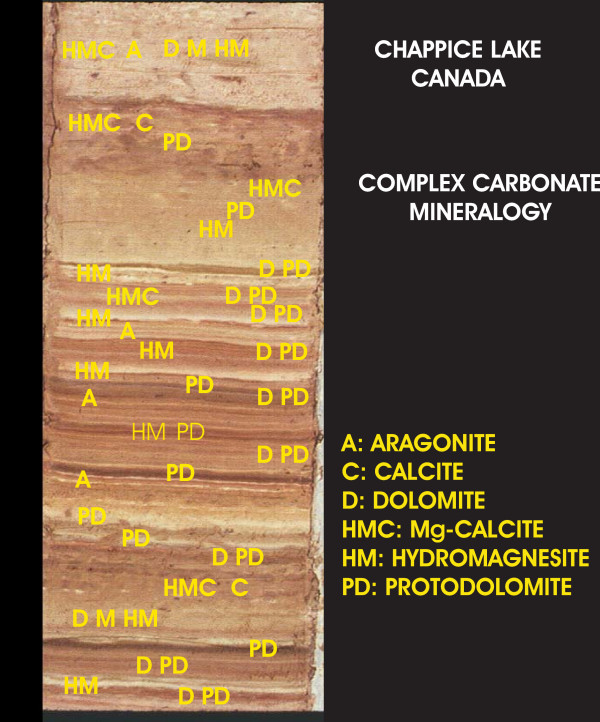
**Chappice Lake laminated carbonates**. Photograph of a section of core from Chappice Lake showing the fine lamination and the complex carbonate mineral assemblage present in this otherwise relatively simple playa basin; modified from [10, 59].

A final example showing the diversity of Mg-carbonate mineral genesis in these lakes is that of Chappice Lake in southeastern Alberta. Chappice Lake is a small, hypersaline, groundwater-fed playa whose brine is dominated by Na and SO_4 _[262-265]. Although endogenic carbonates are not forming in the lake today, the 7000 year long stratigraphic record preserved in the basin contains thick sequences of very finely laminated carbonates [266]. The carbonate crystals and crystal aggregates making up these laminae are euhedral and contain no petrographic evidence of reworking, abrasion, corrosion, or diagenetic alteration, thus suggesting the laminae were generated by inorganic precipitation from within the water column. The lack of detrital grains in these laminae and the absence of rhythmicity indicate relatively rapid and non-annual precipitation events. As shown in Figure 22, it is clear from the detailed mineralogical composition of the sequence that the brine underwent striking compositional changes in both carbonate alkalinity and cation ratios.

**Figure 23:**
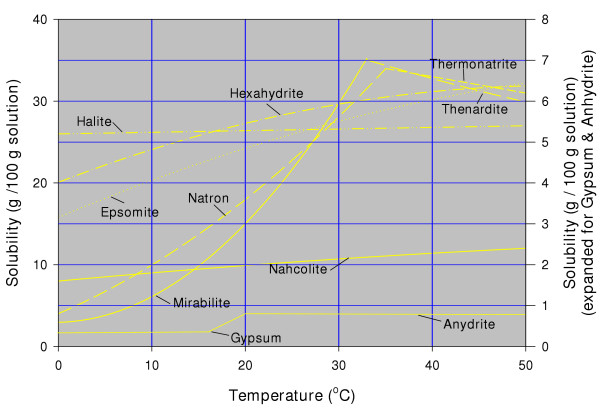
**Temperature-solubility relationships of binary salt systems**. Relationship between temperature and solubility of commonly occurring evaporite minerals in the lakes of the northern Great Plains. Modified from [63].

The mineralogy of the endogenic and authigenic sulfates in the Great Plains' lakes is diverse and the genesis of these inorganic components complex. Not only is the stable sulfate mineral suite controlled by the ionic composition and cation ratios in the brine, but also the temperature at which the precipitation occurs is also very important (Figure 23). For example, in a simple binary salt system (e.g., Na-SO_4_-H_2_O, Mg-SO_4_-H_2_O, or Na-Cl-H_2_O) the solubility of mineral phases such as mirabilite (Na_2_SO_4 _10H_2_0), and epsomite (MgSO_4 _7H_2_O) show enormous ranges over temperatures normally encountered by the brine on a diurnal and seasonal basis. In contrast, common non-sulfate salts, such as halite or bischofite (MgCl_2 _6H_2_O), exhibit comparatively little solubility change over these near-surface temperature ranges. In more complex aqueous systems, such as Na-Mg-Cl-SO_4_-H_2_O, prediction of the stable phase(s) becomes somewhat more complicated, but the influence of temperature remains important.

**Figure 24:**
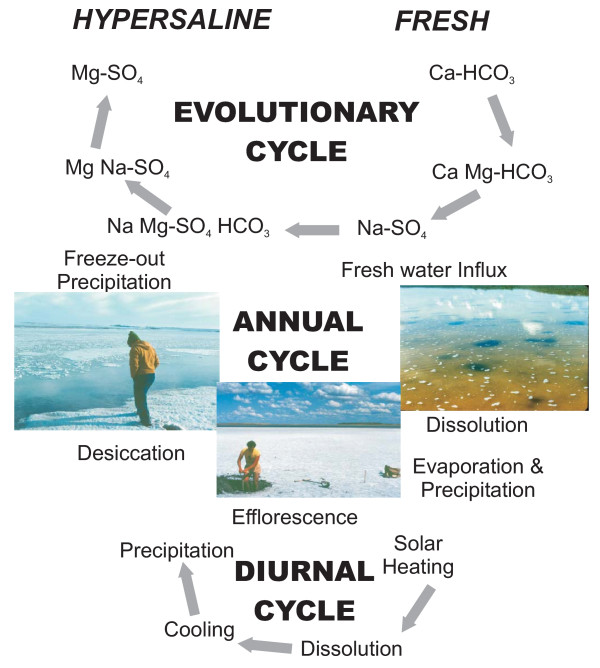
**Sedimentary cycles in cold saline playa lakes of the northern Great Plains**. Schematic illustration showing the major cycles (diurnal, annual, evolutionary) characteristic of playas in the northern Great Plains; modified from [10, 59].

The effect of temperature on the mineral suite in these lakes is most obvious in the playas and shallow lakes/wetlands [219,267,268] where the annual cycle of sediment accumulation (precipitation) and dissolution is readily apparent (Figure 24). However, the deep perennial salt lakes are also strongly influenced by the annual (and diurnal) temperature regime. For example, in Freefight Lake, the surficial waters become supersaturated with respect to mirabilite and epsomite during the winter due to low temperatures of the upper part of the mixolimnion (-10 to -15°C depending on salinity) and the formation of an ice cover which increases the brine concentration in the upper metre. The precipitated Na_2_SO_4 _10H_2_O and MgSO_4 _7H_2_O crystals undergo corrosion and dissolution as they settle through the understaturated (-5 to 5°C) mixolimnion below the ice cover. Dissolution of these sulfate salts in the mixolimnion is of sufficient magnitude to, in turn, affect the temperature of the water. The dissolution of mirabilite is a strongly endothermic reaction. When 1 mol of mirabilite dissolves the reaction absorbs over 4 MJ of heat. This heat must be acquired from the surrounding water, thereby lowering the temperature. However, at the chemocline sulfate salt precipitation begins anew, despite a still higher temperature, due to the greater salinity. Gypsum shows a similar pattern of precipitation from the surficial water; dissolution within the mixolimnion, and precipitation through the monimolimnion, except the supersaturation at the surface is usually brought about during the ice-free season due to evaporative concentration of the brine. Thus, the petrography of the resulting deep-water sulfate salts in this basin records a complex series of multiple precipitation and dissolution events that are controlled largely by seasonal temperature fluctuations within the water column.

### A spectrum of lacustrine sedimentary processes

*"I often say that if you measure that of which you speak, you know something of your subject; but if you cannot measure it, your knowledge is meager and unsatisfactory." *(Lord Kelvin)

The lakes of the northern Great Plains offer an excellent opportunity to examine the processes of lacustrine sedimentation on both a local and regional scale. As is the case with most other depositional settings, these lakes exhibit a continuous spectrum of sedimentary regimes. This continuum exists in terms of nearly every parameter that can be evaluated for the basins: morphology, hydrology, degree of permanence, brine salinity and ionic composition, sediment character, composition, etc. This great diversity presents somewhat of a problem in studying and discussing the lakes.

In order to subdivide this continuum, several "end member" types of lakes can be recognized (Figure 19). A basic sedimentological distinction must be made between the basins whose brines are shallow enough to permit periodic drying and those whose brines are deep enough to maintain a relatively permanent water body. The suite of processes operating in shallow intermittent basins (i.e., playas) includes: cyclic flooding and desiccation of the playa surface, formation of salt crusts, efflorescent crusts, hardgrounds, spring deposits, and intrasedimentary salts, formation of solution pits and chimneys, and periodic detrital sedimentation by sheet flow and wind (Table 3; Figure 24). Most previously published summaries of these basic playa processes deal with lacustrine systems occurring in warm and dry climatic regimes [10,27,199]. The five to six months of subfreezing temperatures experienced in western Canada, as well as the occurrence of snow and high seasonal runoffs associated with snowmelt, dictate some modifications and additional processes be considered when studying the playas of the northern Great Plains.

**Table 3:** Lacustrine sedimentary processes operating in saline lakes of the northern Great Plains; after [10, 117, 158, 178].

**Shallow Water-Shallow Basin & Shallow Water-Deep Basin Lakes**
Cyclic flooding & desiccation
Wind setup
Wind-controlled localization of salt ppt
Aeolian influx
Deflation
Pedogenesis
Intrasedimentary salt precipitation
Formation of subsurface salt cements & hardgrounds
Formation of carbonate crusts
Precipitation of salts at air-water interface
Formation of crystal rafts & aggregates
Evaporative pumping
Formation of efflorescent crusts
Microbialite formation
Formation of vegetation mats
Development of meromixis
Subaqueous cumulate & bottom salt ppt
Formation of salt spring deposits
Formation of rounded accretionary salt grains
Reworking/redistribution of clastic salts
Temperature-induced mineral transformations
Phase changes
Formation of salt cements
Salt karsting
Mud diapirism
Sediment disruption by freeze-thaw
Remobilization & reworking of fine-grained clastics

**Deep Water-Deep Basin Lakes**
Development of meromixis
Bio-mediated carbonate precipitation
Evaporite carbonate precipitation
Formation of subaqueous salt cumulates
Solute concentration by formation of ice
Freeze-out precipitation of salts
Sulfate reduction, sulfide mineral ppt
Cyclic and rhythmic sedimentation
Clay mineral authigenesis
Development of thermal stratification
Turbidity flow, interflow
Flocculation of fine-grained material
Shoreline erosion
Delta sedimentation

The large number of individual playa basins in the Great Plains, the tremendous diversity of chemical and hydrologic settings, and the variable impact of humans are the main factors that have given rise to the complex suite of interrelated sedimentary processes in these lakes. To better understand the relative importance of the many and diverse processes, Q-mode cluster analyses has been applied to help quantitatively group various classes of playas in the region [77]. This analysis identified five groups of playa basins. One group of lakes, typified by the Chaplin, Bigstick, and the Quill Lakes complex, are characterized by having large areas, moderate salinities but high Cl contents, and high detrital to endogenic sediment ratios. Mudflat and sand flat sedimentary processes dominate. In contrast, basins such as Snakehole, Whiteshore, and Muskiki are marked by brines with very high salinities that are almost completely dominated by sodium and sulfate ions. Sedimentation in this group of lakes is controlled largely by chemical processes and the basins contain thick sequences of bedded to massive salts. Chemical sedimentation processes also dominate in lakes such as Ceylon, Ingebright, and Vincent, but they exemplify a third group of playas distinguished by their relatively small areas, and high shoreline length and development indices (i.e., very irregular shorelines or very long, linear basin morphology). Lakes belonging to the final two groups include those whose brines are characterized by calcium, magnesium, and bicarbonate ions rather than sodium and sulfate. Sedimentation is controlled by either mixed chemical/physical processes (e.g., Verlo, Corral, Lydden) or mainly chemical (e.g., Metiskow Lake).

Despite the range of sediment types and hydrology of these lakes, the processes in these basins create a discrete suite of modern sedimentary facies. Although significant variations in the development of these facies do exist from basin to basin, a basic facies pattern common to most of the playas in western Canada can be recognized. The outer shoreline/nearshore complex comprises colluvium, mudflat/sand flat, and beach facies, and grades basinward into a salt pan complex. This basic pattern shares many of the same features recognized in saline lakes in British Columbia [269-272] and is broadly similar to the facies distributions in playas in Spain [273,274].

The colluvium facies consists of an often chaotic mixture of coarse to fine detrital material derived from the adjacent glacial deposits by mass wasting and creep. The mudflats are characterized by a mixture of sandy to silty detrital sediments often capped by thick efflorescent crusts and cemented hardgrounds. Unlike efflorescent crusts on mudflats in playas elsewhere [199,275,276], these crusts are not monomineralic. They usually consist of a complex mineral assemblage of both hydrated and anhydrous Na, Na+Mg, and Ca sulfate and sulfate-chloride salts [277,278,63]. In some basins, carbonate hardgrounds and crusts are also present. The mudflats are areas of active beach processes during seasonally high water levels, but for most of the ice-free season evaporation is the dominant process. Evaporation during the warm summer months results in upward capillary movement of groundwater, ionic concentration, and intrasedimentary mineral precipitation. Once efflorescent crusts have formed, further evaporation is curtailed, and the marginal areas of the lake can maintain high moisture contents throughout the dry season.

**Figure 25:**
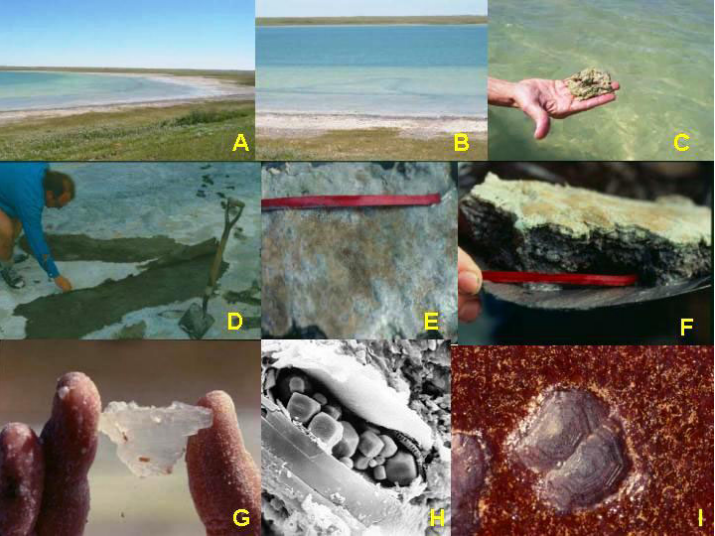
**Microbial mats and other sedimentary-biological features**. Examples of modern bio-sedimentary components of the salt lakes of the northern Great Plains of western Canada. A and B. Modern bio-sedimentary facies in Freefight Lake. Colluvium and mudflats/salt flats grade basinward into a 50 meter wide zone of microbial mats and then into deepwater basinal facies. C. Example of microbial mat material ripped up by wave agitation. D, E, and F. Examples of microbial and vegetation mats from saline lakes in the Great Plains. Note the laminated nature of the sediment immediately below the modern mat in F. Carbonate mineral diagenesis in this environment has created a considerable range and diversity of authigenic species (see also Figure 22). G. Hopper shaped mirabilite crystal incorporating *Artemia *sp. H. Bio-induced early diagenetic pyrite filling a diatom frustrule. I. Hopper shaped salt crystals floating at the surface of a brine pool. The red colouration of the water is due to the high abundance of *Artemia *sp.

The mudflats can be colonized by extensive areas of algae and cyanobacterial mats (Figure 25). The sediments in these areas are distinctively laminated [266], organic-rich, and are usually sites of biogenic carbonate mineral genesis and diagenesis. Many of the more exotic carbonate mineral species found in the Great Plains (e.g., hydromagnesite, psuedohydromagnesite, kutnahorite, siderite, tychite, huntite) have been identified in these biolaminated algal flat sediments. To date, the biology and biogeochemistry of these mats have undergone only cursory examination [207,204,279].

The salt pan complex usually comprises over 50% of a typical playa basin. It can be covered by brine for much of the ice-free period, but is often exposed by late summer. This facies is usually characterized by elevated endogenic to detrital sediment ratios; however, a complete gradation exists from salt-dominated pans to mud-dominated basins. The most distinctive feature of the salt pan facies is its distinctive annual cycle [208,77,68,10]. During spring, relatively dilute inflow from melting snow and rainfall dissolves much of the very soluble salt that may have been precipitated the pan during the previous dry episode. This dissolution results in a significant increase in brine salinity and usually dramatic changes in ion ratios. These brine compositional changes at this time drive many of the penecontemporaneous diagenetic reactions that take place during the initial seasonal flooding of the playa [237,280]. Throughout the rest of the ice-free period brine salinities continue to increase due to evaporation. As discussed above, even minor diurnal temperature changes can cause massive salt precipitation or dissolution. If brine remains in the basin after the onset of freezing conditions, considerable thicknesses of salts can occur due to freeze-out precipitation.

The playa pans can be further subdivided into several subfacies on the basis of mineralogy and crystal morphology [68]. Near the margins of the pan where the salts interfinger with the mudflat facies, a zone of large dog-tooth mirabilite crystals develop, which grow displacively downward into the soft, water-saturated mudflat sediments (Figure 21H). Further out into the center of the basin, the floor of the salt pan usually consists of a mosaic of large, interlocking, bladed crystals. The specific mineralogy in the salt pan is controlled by the ionic content of the brine and the diurnal and seasonal temperature fluctuations experienced. Elevated temperatures favour minerals such as thenardite, hexahydrite, anyhydrite, and thermonatrite whereas cooler temperatures encourage mirabilite, epsomite, natron, and gypsum.

Superposed on these salt pan evaporites or clastics can be zones of *mirabolites *and/or sediments associated with spring openings. Mirabolites are rounded accretionary grains (analogous to carbonate pisolites) composed of Na_2_SO_4 _10H_2_O or MgSO_4 _7H_2_O that form in the shallow, supersaturated, and wind agitated brine (Figure 21J). These mirabolites can be moulded into bedforms, shoals, and ridges on the pan surface or form beach deposits on the marginal mudflats. Spring openings can be sites of massive salt precipitation because these are areas where water of different temperatures and compositions mix (Figure 21K). Frequently the springs continue to discharge onto the playa floor after the pan has been completely desiccated. This continued discharge can form large, low mounds and ridges.

In summary, present-day sedimentation in the playa basins of the Canadian Plains region is controlled by the interplay of: (a) flooding of the playa which causes dissolution of soluble minerals of the salt pan facies and efflorescent crusts of the mudflat and sand flat facies; (b) evaporative concentration of the brine which results in supersaturated conditions and precipitation of various soluble and sparingly soluble salts; (c) detrital influx by streamflow, wind, and spring discharge; and (d) organic productivity. In addition, evaporative pumping of shallow groundwater in the mudflats and sand flats causes growth of intrasedimentary displacive and poikilitic salt crystals in the near-surface clastic sediment.

This suite of interrelated sedimentary mechanisms operating in playa basins of the Great Plains is in contrast to the deposition in the region's perennial lakes, which appears to be controlled by a much smaller array of processes (Table 3). The majority of processes, such as shoreline erosion, beach formation, wave/current distribution of sediments, deltaic sedimentation, and turbidity flow and interflow, are basic sedimentological processes common to any perennial basin. Numerous papers and compilation volumes already provide excellent summaries of these processes in perennial lakes [281,15,282,22], as do most current introductory geoscience textbooks. However, several processes are unique to the perennial basins of western Canada or are of such fundamental importance that they will be introduced here briefly.

Probably the single most critical process operating in the perennial saline lakes is development of stratification of the water column. The influence of temperature stratification on seasonal carbonate mineral saturation and equilibria in the lakes is well known. However, the superposition of temperature stratification on an already chemically stratified water column complicates many mineral precipitation and dissolution reactions. In Deadmoose Lake, for example, both evaporitic and biologically-induced carbonate precipitation occurs in the well-mixed epilimnion. Most of these endogenic carbonates, however, are dissolved upon passing through the thermocline into the anoxic, lower pH water of the hypolimnion, such that the modern lake only contains carbonate-rich muds at water depths of less than 8 m [261,163,117]. In contrast, endogenic CaSO_4 _2H_2_O precipitation occurs below about 12 m depth. This evaporitic mineral is found only in the modern sediments of the monimolimnion. A further complication in the gypsum genesis in this meromictic lake is that the surface waters become supersaturated with respect to CaSO_4 _2H_2_O during the winter and gypsum precipitation occurs. However, sediment trap data indicate that this gypsum originating in mixolimnion is re-dissolved before reaching the chemocline. The same complexity arises for mirabilite: precipitation of this sulfate mineral occurs both at the chemocline and in the surface water during winter. However, it is only preserved in the sediments of the monimolimnion at water depths greater than 20 m. Although this inhomogeneity can be relatively easily modelled once sufficient details are known about the chemical budgets of the stratified brine, any interpretation of the stratigraphic record is made much more obscure because even subtle chemical and/or temperature changes can result in substantial changes in mineral precipitation and preservation.

Two other important aspects of deposition in these perennial lakes of the Great Plains are the generation and accumulation of deep water-soluble salts, and the extraordinarily high rates of sedimentation experienced by some basins. Many of the non-playa lakes in the region are characterized by either mixed endogenic-allogenic sediments or entirely allogenic deposits [157]. However, it has been shown that in some basins soluble and sparingly soluble salts are forming in deep water, offshore environments [283,204,261], [284-286,227,10]. Sedimentologists have recognized for some time that there are very few examples of modern sedimentary environments in which deep water evaporite mineral formation is occurring. This lack of modern deep water settings in which evaporites occur is problematic since many ancient evaporitic sequences have been interpreted as forming in deep water [287-289]. Consequently, the perennial lakes in the Great Plains in which deep water salts are forming today provide a critical analogue in helping to understand the sedimentology, geochemistry, and stratigraphy of these ancient deposits.

To date, three basins in the Great Plains have been identified in which soluble evaporite minerals are forming and accumulating in their deep, offshore areas: Deadmoose, Little Manitou, and Freefight Lakes. It must be emphasized that there are probably numerous such basins among the many perennial lakes of the region, but our knowledge of the deep basinal facies of most of the basins is limited. Furthermore, subaqueous salt precipitation by freeze-out mechanisms have been reported from numerous other lakes in the region [80,175,157] but presumably these salts are seasonal and re-dissolve during the ice-free period.

Both Deadmoose and Freefight Lakes are hypersaline and meromictic, whereas Little Manitou experiences 'temporary' meromixis. All three lakes are dominated by sodium, magnesium and sulfate ions but the mineralogy of the modern offshore precipitates is somewhat different in each lake as controlled by the specific ionic ratios in the brines. As outlined above, the diurnal and seasonal temperature fluctuations of the mixolimnions in Freefight and Deadmoose Lakes and the elevated salinities of the monimolimnions in these basins provide a complex multi-site source for the endogenic evaporite precipitates within the water columns. In Little Manitou, it appears that brine mixing associated with subaqueous spring discharge and the irregular breakdown of the weak meromixis initiates precipitation.

In addition to the formation of these deep water salts, the rate at which they are accumulating is noteworthy. For example, sedimentation rates at the sediment-water interface in the deepest part of Freefight Lake average nearly 30 kg m^-2 ^yr^-1^. While these sediment trap data do not represent *net *accumulation, nonetheless the stratigraphic sequence recovered in the offshore areas of the basin suggest linear accumulation rates in excess of 2 cm yr^-1^, values that are entirely consistent with the extraordinary mass/year sedimentation rates. As discussed elsewhere [290-292], such high rates of chemical sedimentation should be expected in an evaporitic regime. However, until the discovery of these salt lakes in western Canada, such rates were not adequately documented in modern deep water environments.

## Northern Great Plains paleolimnology

*"It has long been my feeling that when a geologist gets into trouble, he changes the climate." *[293]

Until recently, there has been relatively little research effort directed toward investigating the paleohydrology, paleolimnology and paleoenvironmental settings of the saline lakes of the northern Great Plains [294]. There are many reasons for this paucity of study, including difficult chronology problems associated with the widespread abundance of 'old' carbon and its incorporation within the lacustrine deposits [295], the common occurrence of drying/desiccation horizons and incipient soil profiles within the lacustrine stratigraphies [240], and ubiquitous diagenesis and post-depositional physical/chemical alternation of the sediments in the basins [280,158]. However, beginning in the 1990's, the recognition of global change and the necessity to better understand the past climate of the region [296] caused an explosion of interest in the Holocene and late Pleistocene records of the lakes in the Great Plains. The major objective of many of these projects was to decipher the timing and severity of postglacial climatic changes and their geomorphic impact on the prairie landscape [58,297-302]. Clearly, investigation of the Holocene and late Pleistocene stratigraphic records preserved in the lakes of the region forms a pivotal role in accomplishing this objective. Because of their great diversity in morphology, chemistry, and depositional processes, these basins offer an outstanding opportunity to examine past environmental conditions.

Furthermore, the large number of lacustrine basins and their presence over such a broad area will ultimately allow paleolimnologists to integrate numerous sites in a variety of hydrologic and geomorphic settings in order to gain a regional perspective of environmental changes.

**Figure 26:**
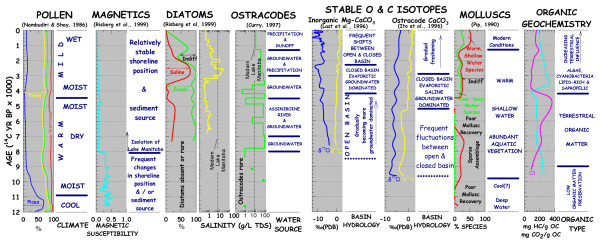
**Lake Manitoba paleolimnology**. Summary diagram showing the paleolimnological interpretations of Lake Manitoba. Modified from [233].

Of the approximately 150 salt lakes for which modern sedimentological data are available, the stratigraphic records of fewer than two dozen have been investigated in any type of detail [294,303,59]. The two deep, meromictic basins east of Saskatoon mentioned previously (Waldsea and Deadmoose Lakes) have received considerable paleolimnological attention. The mid to late Holocene records in these two basins suggest dramatic fluctuations in water levels, organic productivity, and chemical composition [304,305]. Similar changes in brine chemistry and hydrology were interpreted from the stratigraphy of Ceylon Lake in southern Saskatchewan [68,306,307]. The salts deposited in this playa lake suggest that the basin evolved from a relatively low salinity, riverine lake to one in which initially Na-rich and then Mg-rich hypersaline brines dominated. Lake Manitoba, the largest saline lake in the Great Plains, has also undergone intensive paleoenvironmental study [160,236,241,239,233]. Significant changes in water levels during the 12,000-year long history of this lake are associated with brine chemistry changes (particularly with respect to the Mg/Ca ratio of the lake water) and organic productivity fluctuations (Figure 26).

Paleoenvironmental interpretations of salt lake sediments are not without pitfalls. Saline and hypersaline lacustrine environments are amongst the least understood depositional regimes in sedimentary geology. Thus, interpretations of the preserved stratigraphic records are hampered by this incomplete understanding of the modern depositional and diagenetic processes.

Although the relatively few lacustrine sedimentary records that have been examined clearly indicate dramatic changes in water levels and related brine chemistry, the causal mechanism(s) of these temporal changes are still largely unknown. Climate has certainly controlled the sedimentation and geochemistry of nearly all of the basins. However, the precise roles of other factors, such as fluctuating groundwater hydrology and hydrochemistry, or postdepositional alteration of the sediments, still remain to be evaluated.

Despite the sensitivity of these deposits to environmental change, interpreting the records in terms of paleoclimate, hydrology, and chemistry is fraught with difficulty. Factors that complicate these interpretations include: diagenesis of the evaporites, post-depositional physical disruption of the sediments, and a lack of proper understanding of the depositional processes operating in lakes of this type. Furthermore, an active and growing industrial minerals industry based on the deposits of the salt lakes has obliterated, and will likely continue to adversely affect, the stratigraphic records of some of the basins with the greatest research potential. Notwithstanding these problems, the sediments of the salt lakes provide the best and, in some cases, only record of past environmental conditions in this semi-arid region. Paleolimnology in the northern Great Plains is poised for a rapid expansion, fuelled by the combination of significant technological breakthroughs, improvements in methodology, and a more positive view of the importance of paleolimnological research in environmental management.

## Biological processes

*"The absence and incomplete gathering of physiochemical habitat data to associate with biological collections have impeded progress in the classification of distinct ecological communities in salt lakes"*[308]

Biological processes in salt lakes of the Great Plains are overall quite similar to those in shallow, fresh, standing waters, notwithstanding their physical and chemical extremes. Processes such as photosynthesis cause a rise in pH as CO_2 _is utilized by the flora, which, in turn, creates favourable conditions for carbonate precipitation [36]. The biota, however, differ between fresh and saline lakes [309]; at low salinities the species composition of salt lakes is comparable to that of their freshwater counterparts [303]. As salinity increases the diversity of species declines [37], and as salinities reach extremely high values, species diversity becomes very low and the lake is usually dominated only by halotolerant organisms [37]. Ionic composition also affects species diversity. Certain taxa are found in hypersaline waters dominated by a particular solute; anions including chloride, bicarbonate-carbonate and sulfate are important controlling factors in the species composition of salt lakes [308]. Because the brines in many of these lakes are sulfate rich, most of the meromictic lakes in the Great Plains region have a plate of purple sulfate reducing bacteria at the chemocline [175]. The importance and role of these organisms in the saline lake ecosystem requires further investigation, especially with respect to their potentially important role in the formation of some carbonate minerals.

Fossil remains of some organisms of the Great Plains salt lakes have been evaluated for use as paleoindicators. Research on the feasibility of siliceous algae, fossil pigments, as well as ostracodes for use as paleoindicators of changes in salinity and climate have been carried out on some of these lakes [310-312].

## Saline springs

*"...at this rate [of spring discharge] it would have taken only about 5.7 million years to remove all the missing salt from the Devonian in Saskatchewan and Manitoba*." [214]

Springs and spring deposits (travertine, tufa, sinter, etc.) are common in many terrestrial settings in the Great Plains of North America. Most of these occur in association with freshwater lakes and wetlands [313,156,314], although *saline *springs may be an important and recognizable component of the hydrologic budgets of the salt lakes also [80,315,316,69,227,265]. The springs associated with the saline lakes often form impressive platform and pinnacle accumulations of salts up to several meters high, particularly during the winter when the cold temperatures of the lake water cause rapid and massive precipitation of many of the dissolved Mg and Na sulfates [80,176,77,63].

**Figure 27:**
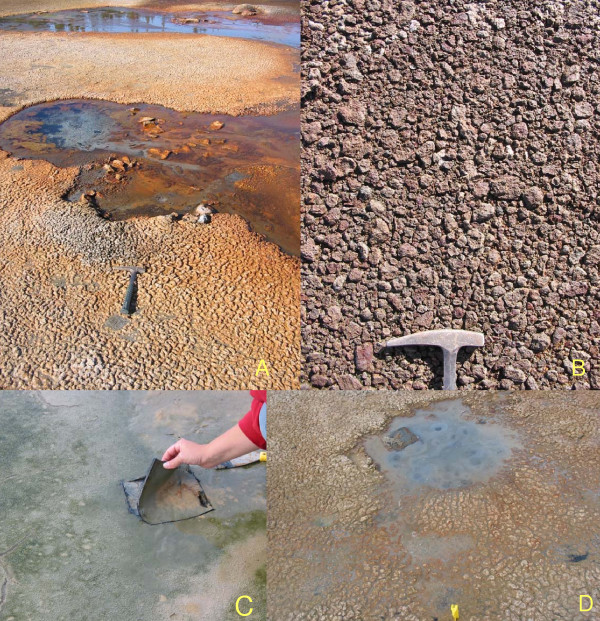
**Saline springs, Lake Winnipegosis region, Manitoba**. A, D: Examples of partially desiccated microbial mat surrounding a saline spring. The brain-like morphology of the mat is coated with evaporitic minerals. B: Fragmented iron-stained carbonate (high-Mg calcite) tufa deposit. C: Example of living microbial mat near a saline spring; mats can reach several centimeters in thickness and are often laminated.

Saline spring systems in the Great Plains that are *not *associated with salt lakes have generally not received much scientific study. Saline springs are common in the Pasquia Hills region of east-central Saskatchewan, in southern and southeastern Alberta, in the Turtle Mountain area of southern Manitoba, and in the Lake Winnipegosis area of west-central Manitoba [317,132,214,319]. The hydrology, geochemistry, and biological characteristics and processes of the brine springs of the Winnipegosis area of Manitoba have been studied in detail [214,320,322-325]. The chemical composition of the brines, dominated by Na and Cl, is strikingly different than that of the saline lakes of the Great Plains and clearly reflects the dissolution of deeply buried Devonian evaporites by groundwater. The occurrence of collapse breccias and structures both within the Paleozoic bedrock in the eastern part of the Prairies and in the overlying Pleistocene sediments further confirms this groundwater dissolution hypothesis. Unfortunately, the composition, genesis and diagenesis of the tufa and sinter deposits of these springs have not been examined in detail, although the diversity of mineralogy and biologically-mediated carbonate precipitation processes are evident (Figure 27).

In addition to their chemistry, the saline pools, marshes and saltpans associated with the brine springs in the Winnipegosis area are noteworthy because they have been found to harbour a variety of marine organisms [320,321,326,327]. The occurrence, and in some cases, dominance, of marine organisms in saline systems thousands of kilometres from the marine environment requires further investigation into dispersal mechanisms and the paleoenvironmental importance of such factors as avian transport and colonization of non-marine habitats by marine organisms.

## Conclusions and future directions

The scientific study of saline lakes in the northern Great Plains is rich with unique research opportunities. Even though the lakes of the region have been studied for over a century, there are still many un-answered questions about source of the salts and the genesis and diagenesis of the inorganic components in these basins. Some of the more noteworthy sedimentary processes and concepts that have not been adequately investigated include: the role of aeolian transport in terms of sediment budgets; anthropogenic activity and its affect on brine composition, water column structure and ecology; and the impact of extreme weather events and climate change on the physical and chemical setting of the basins. Finally, there has been very little application of stable isotope techniques to better understand the water and sediment budgets of these lakes.

Likewise, since the 1930's there has been considerable effort made to document the biological characteristics and processes operating in selected basins, nonetheless there is still a major gap in our knowledge about the ecological communities of these salt lakes. Similarly, the precise role that the microbiota play in the formation of endogenic and authigenic minerals in the lakes remain largely unexplored. Of particular importance over the coming years is to better decipher how sulfate reducing bacteria and cyanobacteria, which are commonly found in both the deeper and chemically stratified lakes, as well as the microbial mats found in many of these lakes, interact with the inorganic components to help control penecontemporaneous diagenesis and authigenic mineral formation.

Because of the great diversity in basin types, brine chemistries, and depositional processes, the sediments in these lakes offer a tremendous opportunity to examine past hydrological and environmental conditions in the region. The past decade has witnessed considerable growth in interest and research on the Holocene stratigraphic records in these saline basins. It is now recognized that these lakes provide nearly the only source of detailed, high resolution, physical, biological and chemical paleoenvironmental information for the Holocene of the region. Despite the sensitivity of these deposits to environmental change, interpreting the records in terms of paleohydrology, chemistry, and climate is fraught with difficulty. Factors that complicate these interpretations include: diagenesis of the evaporites, post-depositional physical disruption of the sediments, and a lack of proper understanding of the depositional processes operating in lakes of this type.
